# Effects of exercise training on depression, anxiety, and physical health-related quality of life in end-stage renal disease patients receiving maintenance hemodialysis: a systematic review and meta-analysis of randomized controlled trials

**DOI:** 10.3389/fpubh.2026.1788845

**Published:** 2026-04-23

**Authors:** Jiahao Li, Yuanyuan Li, Wanqi Shi, Qianqian Guo, Qian Yang, Zijing Chu

**Affiliations:** 1 The First Hospital of Jilin University, Changchun, China; 2 School of Nursing, Jilin University, Changchun, China; 3 The First Affiliated Hospital of Zhengzhou University, Zhengzhou, China

**Keywords:** anxiety, depression, exercise therapy, maintenance hemodialysis, quality of life

## Abstract

**Background:**

Maintenance hemodialysis patients commonly have adverse emotional states of depression and anxiety, leading to a serious decline in their quality of life. Exercise therapy acts as a supplementary measure that has the potential to relieve negative emotions. However, gaps remain in the current literature.

**Objective:**

To explore the effect of exercise intervention on depression, anxiety, and physical health-related quality of life in maintenance hemodialysis patients, and analyze the influence of population characteristics and intervention plans on the curative effect of exercise therapy.

**Methods:**

A literature search was conducted across 12 databases from inception to November 14, 2025. Two researchers independently screened literature and extracted data based on PICOS framework. R software and Stata software were utilized for data analysis to evaluate intervention effects by calculating standardized mean differences (SMD) and 95% confidence intervals (CI). Sensitivity analysis was done by the leave-one-out method. Egger’s test and trim-and-fill method were used to explore potential publication bias. The revised Cochrane risk-of-bias tool was used to assess the methodological quality of included studies, and the GRADE method assessed the overall quality of evidence.

**Results:**

A total of 27 studies involving 1,597 participants were included. Meta-analysis results demonstrated that exercise improved depression and physical health-related quality of life in maintenance hemodialysis patients, while its effect on anxiety remains uncertain. Subgroup analyses indicated that exercise therapy yielded better outcomes in patients under 60 years of age. Intervention durations exceeding 24 weeks, the adoption of combined aerobic and strength training protocols, and a total weekly exercise volume of more than 120 min were associated with significant improvements in depression. Implementing exercise therapy with specialized equipment could better enhance physical health-related quality of life.

**Conclusion:**

The findings of this study demonstrate that exercise significantly ameliorates depression and modestly improves physical health-related quality of life in maintenance hemodialysis patients. However, due to the small number of included studies, high statistical heterogeneity, and limited assessment of publication bias, the ameliorating effect on anxiety cannot be definitively confirmed, necessitating future large-scale and rigorously designed randomized controlled trials for further verification. Furthermore, this study revealed that the intervention effects are moderated by variables including age, equipment utilization, exercise type, intervention duration, and total weekly exercise volume. Future research should prioritize the development of tailored exercise prescriptions for hemodialysis patients while concurrently enhancing the methodological rigor of study designs. Healthcare professionals should pay attention to improving the mental health of this group by implementing exercise therapy.

**Systematic review registration:**

https://www.crd.york.ac.uk/PROSPERO/view/CRD420251196880, Identifier: CRD420251196880.

## 1 Introduction

According to data from the Global Burden of Disease Study, approximately 780 million people worldwide were affected by chronic kidney disease (CKD) as of 2023. CKD has emerged as a major global public health burden, and its prevalence continues to rise due to the intensifying aging of the population and the increasing incidence of underlying conditions such as diabetes and hypertension (1). Notably, due to the insidious onset and progressive pathophysiological nature of renal injury, a substantial number of patients fail to detect physical abnormalities during disease progression (2). Coupled with unavoidable renal injury factors in daily life (3), the high prevalence of CKD is gradually translating into a high incidence of end-stage renal disease (ESRD) (4). For patients whose condition has progressed to ESRD, maintenance hemodialysis (MHD) serves as the core and primary therapeutic measure for alleviating the condition and prolonging survival (5, 6). However, although advancements in dialysis technology have significantly prolonged patient survival, with the shift in the paradigm of clinical disease management, the goal of healthcare professionals is no longer solely limited to extending life, but increasingly emphasizes improving the patient’s quality of life (7).

Psychological distress, particularly depression and anxiety, is highly prevalent among patients undergoing maintenance hemodialysis. Reports indicate prevalence rates ranging from 39.3 to 68.9% for depression and 36.8 to 45.7% for anxiety, figures that significantly exceed those observed in the general population (8–10). These psychiatric symptoms transcend mere emotional fluctuations and exert profound adverse effects on both psychological and physiological well-being. Psychologically, negative mental states foster pessimism and low mood, significantly compromising treatment adherence (11). Physiologically, MHD patients already sustain a state of chronic inflammation. Emotional disorders further exacerbate this condition by triggering the hypothalamic–pituitary–adrenal (HPA) axis and the sympathetic nervous system, leading to immune dysregulation, intensified inflammatory responses, and heightened susceptibility to infection (12). The progression of inflammation suppresses central appetite and accelerates energy metabolism, thereby inducing Malnutrition-Inflammation Complex Syndrome (MICS). This syndrome contributes to erythropoietin hyporesponsiveness and increased cardiovascular atherosclerosis (13). Ultimately, the synergistic burden of these psychological and physiological factors significantly impairs quality of life and increases mortality risk (14, 15).

Currently, the management of negative emotions in MHD patients primarily involves pharmacological and psychological interventions. However, given the severe renal impairment in MHD patients, pharmacokinetics are altered and drug clearance is significantly reduced (16, 17). Consequently, the selection, dosage, and administration of medications require rigorous consideration (18). Furthermore, the potential side effects and adverse reactions resulting from drug–drug interactions can inflict substantial harm on patients (19), rendering pharmacotherapy highly challenging. Although professional psychotherapy has been proven effective in alleviating psychological distress, its accessibility is frequently constrained by high costs and a shortage of qualified mental health professionals (20, 21). Additionally, MHD patients often experience stigma and may be reluctant to disclose their true inner feelings to healthcare professionals (22). Therefore, exercise therapy is increasingly recognized as a safe and accessible adjunctive strategy for preventing and mitigating mental health issues in this population. Exercise therapy is defined as a planned physical activity program designed to achieve specific therapeutic goals, executed regularly using equipment or body weight to restore physical function and alleviate disease burden. With the continuous development of exercise therapy, some literature indicates that regular exercise training serves as a promising nonpharmacological intervention, which has the potential to mitigate the psychological symptom burden in MHD patients. Moreover, structured exercise training serves as a critical intervention for counteracting sarcopenia and functional frailty (23). The combined improvements in psychological and physiological domains generate a synergistic effect that significantly enhances the quality of life for patients.

The specific impact of exercise on mental health outcomes within the context of maintenance hemodialysis remains insufficiently explored, and gaps remain in the current literature regarding the optimal exercise modalities, intervention duration, and the specific influence of patient age and equipment utilization. To address these research gaps, we conducted the present meta-analysis. Randomized controlled trials (RCTs) constitute the highest level of evidence for establishing the efficacy of clinical interventions (24). In this meta-analysis of RCTs, we evaluated the efficacy of exercise therapy in reducing the severity of depression and anxiety, as well as enhancing physical health-related quality of life among MHD patients. Furthermore, we investigated the effects of exercise therapy across different age groups and analyzed the significance of using specialized equipment. These aspects represent directions that have not been addressed in previous studies. This study aims to provide valuable insights into exercise therapy as an adjunctive intervention to improve physical and mental outcomes in MHD patients, thereby assisting healthcare professionals in delivering personalized and precise exercise guidance.

## 2 Methods

### 2.1 Study protocol

This systematic review was reported in accordance with the Preferred Reporting Items for Systematic Reviews and Meta-Analyses (PRISMA) guidelines (25). The study protocol was prospectively registered with the International Prospective Register of Systematic Reviews (PROSPERO) on November 8, 2025, prior to the initiation of the formal literature search, under the registration number CRD420251196880. As this review relied exclusively on data from previously published randomized controlled trials that had already secured approval from their respective institutional review boards and informed consent from participants, neither separate ethical approval nor informed consent was required for the current study.

### 2.2 Data sources and search strategy

A comprehensive literature search was conducted across PubMed, Excerpta Medica Database (Embase), The Cochrane Library (Cochrane Central Register of Controlled Trials, CENTRAL), Web of Science (Core Collection), Cumulative Index to Nursing and Allied Health Literature (CINAHL), MEDLINE, Scopus, ProQuest, Chinese Biomedical Literature Database (CBM), Chinese National Knowledge Infrastructure (CNKI), China Science and Technology Journal Database (VIP), and Wanfang Database. The search covered the period from inception through November 14, 2025. To minimize the risk of omitting relevant studies, we also manually screened the reference lists of all included articles. No language restrictions were imposed. The search strategy employed a combination of Medical Subject Headings (MeSH) and free-text terms, including “exercise,” “hemodialysis,” “depression,” “anxiety,” and “quality of life”. Detailed search strategies are presented in Supplementary Table 1.

### 2.3 Inclusion and exclusion criteria

The inclusion criteria were established based on the PICOS framework, comprising participants, interventions, controls, outcomes, and study design: (1) Participants were adults aged 18 years or older who had been undergoing maintenance hemodialysis for at least 3 months; (2) Interventions utilized any modality of exercise, including aerobic training, strength training, combined aerobic and strength training, respiratory training, or mind–body exercise; (3) The control group received usual care, maintained a sedentary lifestyle, performed sham exercises, or received health education; (4) The reported outcomes included at least one of the following: depression, anxiety, or quality of life; (5) The study design was restricted to RCTs. Studies were excluded if they met any of the following criteria: (1) ineligible study design, such as non-randomized controlled trials or conference abstracts; (2) ineligible population; (3) ineligible intervention; (4) irrelevant outcome; (5) the data were insufficient for extraction or synthesis; or (6) the full text remained unavailable even after contacting the corresponding authors.

### 2.4 Study selection and data extraction

After the database search, researchers imported the retrieved records into the reference management software EndNote 21 and removed duplicate records. Two researchers independently screened the titles and abstracts of the retrieved records strictly according to the established inclusion and exclusion criteria. Subsequently, they downloaded and carefully read the full texts of the studies that passed the initial screening to determine the studies that finally met the inclusion criteria. On the other hand, researchers also re-checked the reference lists of the included studies to discover other potentially eligible reports. If disagreements appeared during the screening process, they were resolved by negotiation between the two researchers, or by consulting a third researcher to decide the final result.

After the screening process ended, two researchers used a pre-designed Excel spreadsheet to extract data. This spreadsheet comprehensively covered relevant information of each study, including: research publication details, study population characteristics, specific content of intervention measures, outcome indicators, and measurement methods. If there was missing data or incomplete information in the included studies, researchers contacted the original authors for additional information. A third researcher was responsible for cross-checking the extracted data, and if data differences were found, the final result was determined through joint discussion.

### 2.5 Quality assessment

Two researchers independently assessed the risk of bias in the included RCTs using the revised Cochrane risk-of-bias tool for randomized trials (RoB 2) and generated risk-of-bias plots (26). The assessment covered the following domains: (1) randomization process; (2) deviations from intended interventions; (3) missing outcome data; (4) measurement of the outcome; (5) selection of the reported result; (6) and overall risk of bias. The risk of bias was categorized into three levels: low risk, some concerns, and high risk. Additionally, the Grading of Recommendations Assessment, Development, and Evaluation (GRADE) framework was employed to appraise the certainty of the evidence (27). This evaluation considered five domains: (1) risk of bias; (2) inconsistency; (3) indirectness; (4) imprecision; (5) and publication bias. The quality of evidence was classified into four levels: high, moderate, low, and very low.

### 2.6 Data analysis

Data analyses in this study were performed using R software (version 4.5.1) and Stata software (version 18.0). Statistical significance was defined as a two-sided *p*-value < 0.05 unless otherwise specified. Given the variability in outcome measurement tools across studies, the standardized mean difference (SMD) was utilized to synthesize the data. Due to the heterogeneity in patient characteristics and specific exercise intervention protocols among the included studies, we selected a random-effects model to pool the data, thereby providing a more conservative and robust estimate of the effect size. To evaluate the robustness of the pooled SMD, sensitivity analysis was performed using the leave-one-out method. Heterogeneity among the included studies was assessed using the I^2^ statistic and Cochrane’s Q test. Heterogeneity levels were categorized based on I^2^ values: less than 30% indicated low heterogeneity, 30 to 60% indicated moderate heterogeneity, and greater than 60% indicated substantial heterogeneity. For the Q test, a *p*-value < 0.10 was considered indicative of statistical heterogeneity. Subgroup analysis and meta-regression were conducted to explore the moderating effects of various variables on the intervention outcomes. Subgroup analyses were conducted based on participant characteristics and intervention protocols, specifically: age, geographical region, type of exercise, intervention duration, outcome assessment tools, timing of intervention delivery, type of control, the utilization of specialized equipment, exercise load progression, and total weekly exercise volume. For multi-arm studies, following the recommendations of the Cochrane Handbook for Systematic Reviews of Interventions, we divided the sample size of the shared control group equally among the comparisons to avoid double-counting control participants and inflating the study’s weight. Publication bias was assessed quantitatively using Egger’s test and qualitatively through visual inspection of funnel plot symmetry. A *p*-value < 0.05 or observed asymmetry in the funnel plot suggested the presence of publication bias (28). Where publication bias was suspected, the trim-and-fill method was employed to adjust for potential bias (29). This technique imputes missing studies to restore funnel plot symmetry and subsequently recalculates the pooled effect size.

## 3 Results

### 3.1 Study results and selection

**Figure 1:**
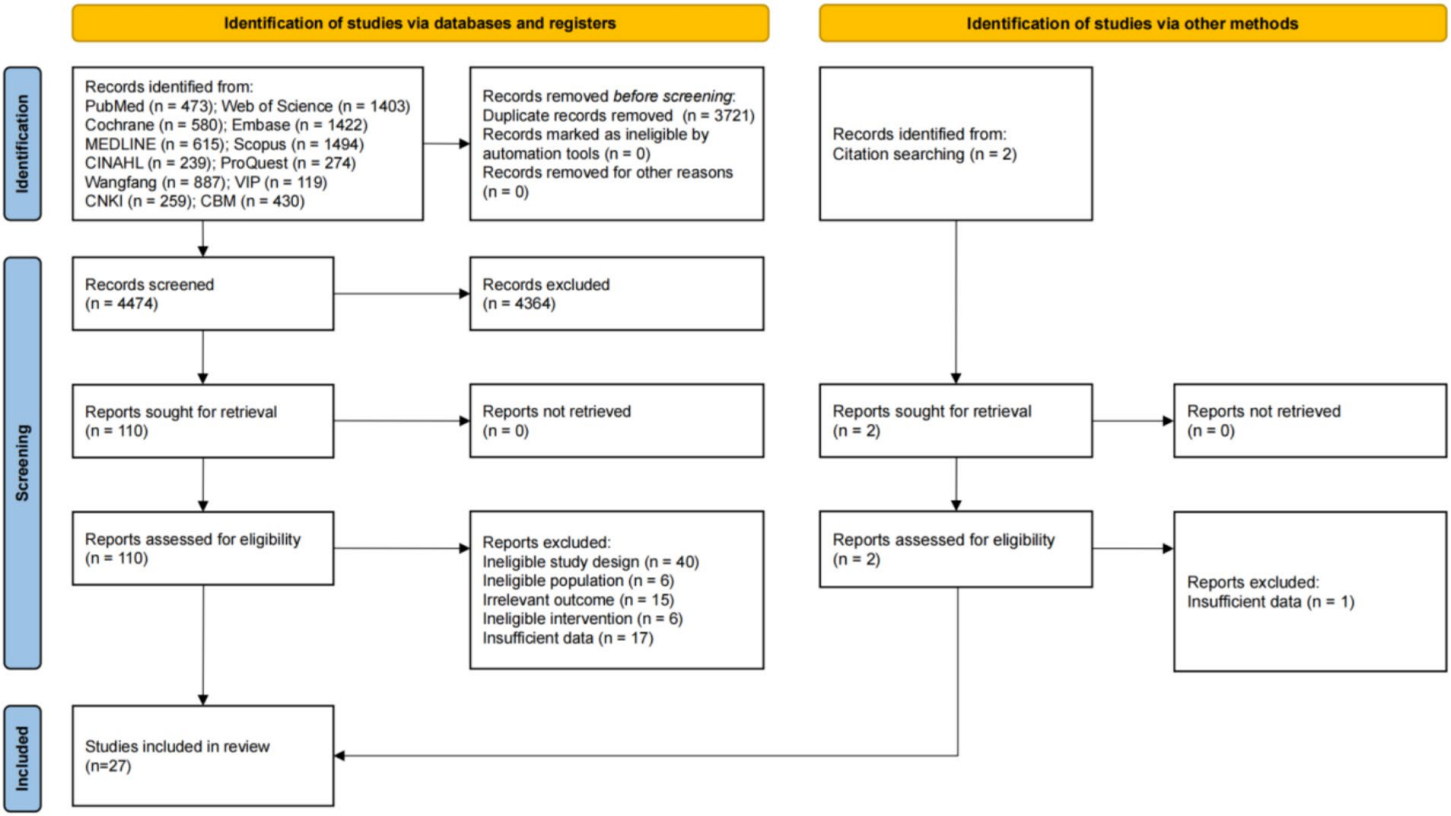
PRISMA flow diagram of study selection.

After searching 12 digital databases, the initial search obtained a total of 8,195 records. Among them, 3,721 duplicate records were excluded by using EndNote 21 software, and the remaining 4,474 records entered the title and abstract screening stage. After carefully reading titles and abstracts, we excluded 4,364 records that did not meet the inclusion criteria. At the same time, we additionally identified 2 records by checking references, and a total of 112 reports were considered to potentially match this study and needed full-text review. Following the full-text assessment, an additional 85 reports were excluded. The reasons for exclusion included ineligible study design (*n* = 40), ineligible population (*n* = 6), ineligible intervention (*n* = 6), irrelevant outcome (*n* = 15), and insufficient data (*n* = 18). The excluded reports and their corresponding reasons for exclusion are detailed in Supplementary Table 2. Finally, 27 eligible studies met the inclusion criteria of this study and were included in the final analysis. The PRISMA study selection flow chart is shown in Figure 1.

### 3.2 Study characteristics

The characteristics of the included studies are summarized in Table 1. The 27 included studies were published between 1997 and 2025. Among the eligible studies, 8 were conducted in China (30–37), 5 were conducted in Brazil (38–42), 5 were conducted in Greece (43–47), 2 were conducted in Korea (48, 49), and 2 were conducted in Iran (50, 51). The United States (52), Czech (53), Netherlands (54), Poland (55), and Thailand (56) each had 1 study.

**Table 1:** The characteristics of included studies.

Study	Country	Sample size		Female (%)	Age (mean)	Setting	Characteristics		Duration (weeks)	Weekly training frequency	Session length (minutes)	Assessment tool
		Intervention	Control				Intervention	Control				
Chen et al., 2010 (52)	US	22	22	47.7	69	Patients were recruited from Tufts Medical Center and Caritas St. Elizabeth’s Medical Center in Boston, Massachusetts	Strength	Sham exercise	24	2	35–50	QOL: SF-36
Deus et al., 2021 (38)	Brazil	81	76	45.2	66.81	Patients were recruited from the affiliated hospitals of universities in Brazil	Strength	Usual care	24	3	60	Depression: BDI
Dobsak et al., 2012 (53)	Czech	11	10	61.9	59.1	Patients were recruited from St. Anna Faculty Hospital in Brno	Aerobic	Usual care	20	3	30–60	QOL: SF-36
Feng et al., 2025 (30)	China	28	30	36.2	56.8	Patients were recruited from Shanghai Sixth People’s Hospital	Strength	Usual care	12	3	30–120	Depression: HAMD Anxiety: HAMA
Fu et al., 2021 (31)	China	86	88	33.9	53.5	Patients were recruited from the 971st Hospital of the Chinese People’s Liberation Army Navy	Mind–body	Usual care	26	3–5	30–60	Depression: SDS Anxiety: SAS
Giannaki et al., 2013 (43)	Greece	12	12	29.2	58.6	Patients were recruited from the University Hospital of Larissa and the General Hospital of Trikala in the region of Thessaly	Aerobic	Sham exercise	26	3	45	Depression: SDS
Huang et al., 2020 (32)	China	16	16	28.1	40.7	Patients were recruited from The First Affiliated Hospital of Xi’an Jiaotong University in Xi’an, Shaanxi Province	Aerobic+Strength	Sham exercise	24	3	30	QOL: SF-12
Jamshidpour et al., 2020 (50)	Iran	15	13	28.6	61.9	Patients were recruited from Milad Hospital in Tehran	Aerobic+Strength	Usual care	8	3	25–55	QOL: SF-36
Kim et al., 2023 (48)	Korea	18	21	48.7	57.1	Patients were recruited from CHA Bundang Medical Center, CHA University	Aerobic	Education	12	3	40–70	QOL: SF-36
Kouidi et al., 1997 (44)	Greece	20	11	51.6	50.7	Patients were recruited from the Renal Unit of AHEPA Hospital in Thessaloniki	Aerobic	Sedentary	26	3–4	90	Depression: BDI
Kouidi et al., 2010 (45)	Greece	24	20	40.9	46.1	Patients were recruited from the Renal Unit of AHEPA Hospital in Thessaloniki	Aerobic+Strength	Usual care	52	3	60–90	Depression: BDI Anxiety: HADS
Li et al., 2025 (71)	China	29	30	30.5	44.7	Patients were recruited from Taihe County People’s Hospital in Fuyang, Anhui Province	Aerobic+Strength	Education	12	3	40	Depression: SDS Anxiety: SAS
Li et al., 2024 (34)	China	40	20	26.7	49.3	Patients were recruited from the First Affiliated Hospital of Nanjing Medical University in Nanjing, Jiangsu Province	Aerobic respiratory	Usual care	12	3	40	QOL: SF-36
Liu et al., 2023 (35)	China	42	42	51.2	57.7	Patients were recruited from Songjiang District Central Hospital in Shanghai	Aerobic	Usual care	24	3	30	Depression: SDS
Marieke et al., 2005 (54)	Netherlands	53	43	34.4	54.7	Patients were recruited from the Groningen Dialysis Center in The Netherlands	Aerobic+Strength	Usual care	12	2–3	60–90	Depression: SDS
Maynard et al., 2019 (39)	Brazil	20	20	45	46.4	Patients were recruited from the Clinic of Nephrology of Sergipe in Brazil	Aerobic+Strength	Usual care	12	3	30–60	Depression: CES-D QOL: SF-36
Ouzouni et al., 2009 (46)	Greece	19	14	18.2	48.7	Patients were recruited from the Renal Unit of AHEPA Hospital in Thessaloniki	Aerobic+Strength	Usual care	43	3	60–90	Depression: BDI QOL: SF-36
Pereira et al., 2022 (40)	Brazil	40	40	37.5	70.3	Patients were recruited from a hemodialysis center in Belem, Para	Aerobic	Education	13	3	30	QOL: SF-12
Prestes et al., 2025 (41)	Brazil	52	26	43.6	57.5	Patients were recruited from Premium Nephrology Clinic in Brasilia and the Clinical Group Home Dialysis Center in São Paulo	Strength	Usual care	24	3	80	Depression: BDI
Rezaei et al., 2015 (51)	Iran	25	26	31.4	43.3	Patients were recruited from Emam Reza Hospital in Kermanshah	Strength	Usual care	10	3	35	Depression: BDI
Rosa et al., 2018 (42)	Brazil	28	24	32.7	55.7	Patients were recruited from the Hemodialysis Center of Bauru State Hospital in Bauru, São Paulo	Strength	Sham exercise	12	3	40–50	QOL: SF-36
Samara et al., 2016 (47)	Greece	15	12	11.1	48.3	Patients were recruited from major nephrology clinics in Thessaloniki	Aerobic	Sedentary	17	3	60	QOL: SF-36
Siou-Hung et al., 2015 (36)	China	32	25	50.9	63.2	Patients were recruited from Wan Fang Hospital, Taipei Medical University, Taipei, Taiwan	Respiratory	Usual care	4	2	30	Depression: BDI QOL: SF-36
Song and Sohng, 2012 (49)	Korea	20	20	50	53.3	Patients were recruited from Uijeongbu St. Mary’s Hospital, The Catholic University of Korea	Strength	Usual care	12	3	30	QOL: SF-36
Turon-Skrzypinska et al., 2023 (55)	Poland	39	46	31.8	60.3	Patients were recruited from the Clinic of the Department of Nephrology, Transplantology and Internal Medicine, Pomeranian Medical University in Szczecin	Aerobic	Usual care	13	3	20	Depression: BDI Anxiety: GAD
Yu et al., 2021 (37)	China	29	30	50.8	53.2	Patients were recruited from Northern Jiangsu People’s Hospital in Jiangsu Province	Aerobic+Strength	Usual care	26	3	60	QOL: SF-36
Yuenyongchaiwat et al., 2021 (56)	Thailand	23	21	40.9	52.1	Patients were recruited from Thammasat University Hospital and Karunvej Hospital in Pathum Thani Province	Respiratory	Sham exercise	8	3	25	QOL: SF-12

BDI, beck depression inventory; CES-D, Center for Epidemiologic Studies Depression Scale; GAD, Generalized Anxiety Disorder Scale; HADS, Hospital Anxiety and Depression Scale; HAMA, Hamilton Anxiety Rating Scale; HAMD, Hamilton Depression Rating Scale; QOL, Quality of Life; SAS, Self-Rating Anxiety Scale; SDS, Self-Rating Depression Scale; SF-12, Short Form 12 Health Survey; SF-36, Short Form 36 Health Survey.

This systematic review covered a total of 1,597 MHD patients, of which 839 were from the exercise intervention group and 758 were from the control group. The sample size of the included studies ranged from 21 (53) to 174 (31). The proportion of women in the study population ranged from 11.1% (47) to 61.9% (53), and the average age of the study population ranged from 40.7 (32) to 70.3 (40) years. All participants in the included studies were recruited in hospitals or dialysis centers.

The exercise intervention measures of the included studies showed diversity in type, frequency, duration, implementation timing, and method. 9 studies carried out aerobic training (34, 35, 40, 43, 44, 47, 48, 53, 55), 7 studies carried out strength training (30, 38, 41, 42, 49, 51, 52), 8 studies conducted combined aerobic and strength training (32, 33, 37, 39, 45, 46, 50, 54), 3 studies adopted respiratory training (34, 36, 56), and 1 study performed mind–body exercise (31). The implementation methods of different types of training were different. Aerobic training was mainly performed through cycling, jogging or walking, and swimming. Strength training stimulated limbs or core muscle groups through elastic bands, dumbbells, or other resistance form activities. Respiratory training was carried out by using sandbags to give a certain load to respiratory muscles. Mind–body exercise required participants to perform traditional Chinese medicine Qigong training to improve body and mind at the same time. It should be reminded that the study by Li et al. (34) set up two intervention groups, which were the aerobic training group and the respiratory training group. The intervention duration of the included studies ranged from 4 weeks (36) to 52 weeks (45), intervention frequency covered twice a week (36, 52) to 3–5 times a week (31), and single intervention duration ranged from 20 (55) to 30 min to 2 h (30). Regarding timing, 8 studies were conducted during non-intradialytic periods (31, 36, 38, 41, 44, 47, 49, 51), 1 combined intradialytic and non-intradialytic exercise (54), and the remaining studies were implemented intradialytically. All interventions were supervised by physicians, nurses, or rehabilitation therapists. In terms of control conditions, 5 studies employed sham exercise (32, 42, 43, 52, 56), 3 provided health education (33, 40, 48), 2 maintained a sedentary lifestyle (44, 47), and the remainder received usual care. Detailed descriptions of the interventions are provided in Supplementary Table 3.

In terms of outcome evaluation, the included studies used different scales to evaluate the influence of exercise intervention on depression, anxiety, and physical health-related quality of life. Regarding the assessment of depression, 8 studies used the Beck Depression Inventory (BDI) (36, 38, 41, 44–46, 51, 55), 5 studies used the Self-Rating Depression Scale (SDS) (31, 33, 35, 43, 54), 1 study used the Center for Epidemiologic Studies Depression Scale (CES-D) (39), and 1 study used the Hamilton Depression Rating Scale (HAMD) (30). Regarding the assessment of anxiety, 1 study used the Hospital Anxiety and Depression Scale (HADS) (45), 2 studies used the Self-Rating Anxiety Scale (SAS) (31, 33), 1 study used the Hamilton Anxiety Rating Scale (HAMA) (30), and 1 study used the Generalized Anxiety Disorder Scale (GAD) (55). Regarding the assessment of physical health-related quality of life, 3 studies used the Short Form 12 Health Survey (SF-12) (32, 40, 56), and the remaining studies used the Short Form 36 Health Survey (SF-36). It needs to be explained that we selected the data of the physical component summary (PCS) domain in SF-36 and SF-12 scales to combine, in order to evaluate the effect of exercise on physical health-related quality of life in MHD patients.

### 3.3 Risk of bias and evidence certainty

**Figure 2:**
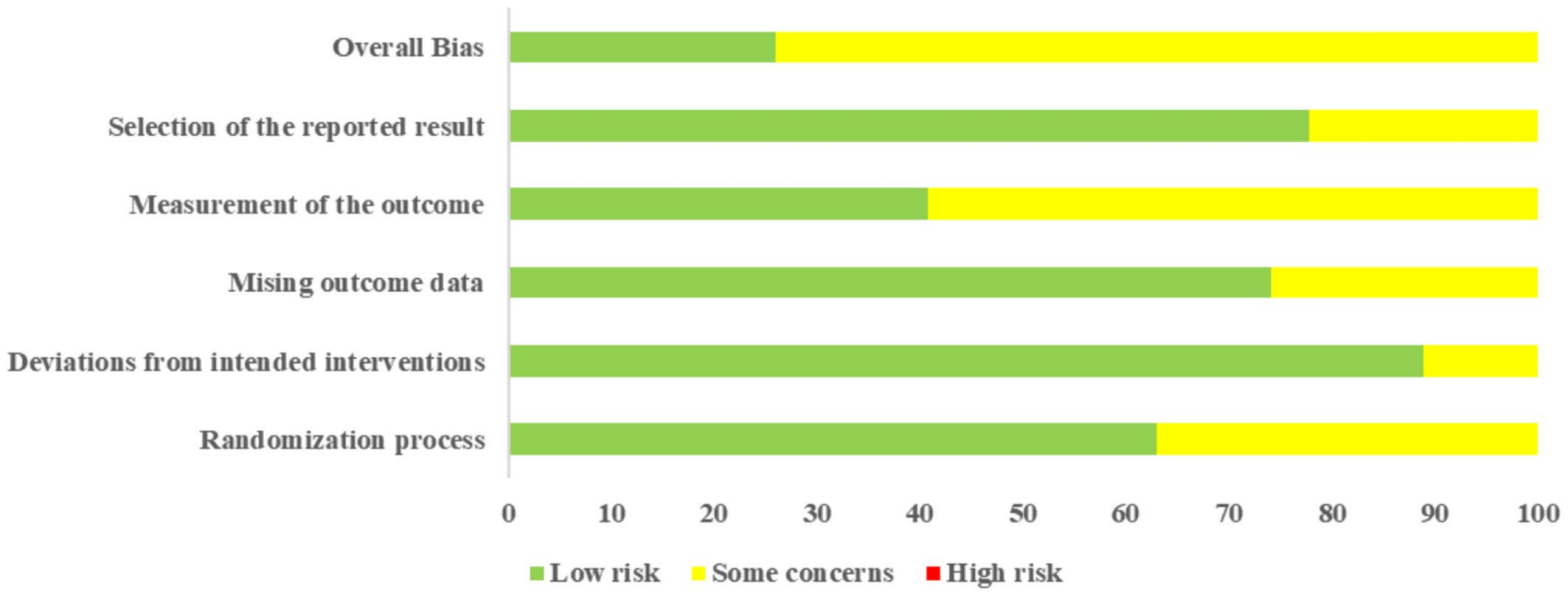
The results of risk of bias assessment of included studies.

Two researchers independently used RoB2 to evaluate the methodological quality of the included studies. Among the 27 included RCTs, all studies reported randomization methods, but 10 studies did not report detailed allocation concealment schemes. 3 studies did not mention whether the research environment caused deviations from the intended interventions. 7 studies might have missing outcome data. 16 studies did not clearly report whether the outcome was measured by assessors who did not know the grouping, which has a risk of subjectively exaggerating the intervention effect. 6 studies did not report clinical trial registration protocols or detailed analysis plans, so there may be selective reporting bias. In short, regarding the overall risk of bias, 7 studies were rated as “low risk” (30, 32, 36, 43, 45, 49, 56), and the remaining studies were rated as “some concerns’’ (Figure 2). Detailed assessment results are available in Supplementary Figure 1.

We performed result summary analysis using the Grading of Recommendations Assessment, Development and Evaluation system software (GRADEpro GDT) according to the relevant guidelines of the GRADE handbook. The summary results show that the quality of evidence related to depression, anxiety, and physical health-related quality of life was judged as moderate, low, and low, respectively. The specific reasons for the assessment and detailed information can be seen in Table 2.

**Table 2:** GRADE evidence certainty.

Certainty assessment							No. of patients		Effect		Certainty
No. of studies	Study design	Risk of bias	Inconsistency	Indirectness	Imprecision	Other considerations	Intervention	Control	Relative (95% CI)	Absolute (95% CI)	
Depression											
15	Randomised trials	Serious^a^	Not serious	Not serious	Not serious	None	562	509	-	SMD 0.79 SD lower (1.01 lower to 0.56 lower)	⨁⨁⨁◯ Moderate^a^
Anxiety											
5	Randomised trials	Serious^a^	Serious^b^	Not serious	Not serious	None	206	214	-	SMD 0.82 SD lower (1.2 lower to 0.45 lower)	⨁⨁◯◯ Low^a,b^
Physical health-related quality of life											
15	Randomised trials	Serious^a^	Not serious	Not serious	Not serious	Publication bias strongly suspected^c^	348	308	-	SMD 0.46 SD higher (0.25 higher to 0.67 higher)	⨁⨁◯◯ Low^a,c^

CI, confidence interval; SMD, standardized mean difference.

^a^Insufficient reporting of outcome assessor blinding raises bias risks for subjective outcomes.

^b^High heterogeneity cannot be fully explained.

^c^The test results suggest the existence of publication bias.

### 3.4 Efficacy of the intervention

#### 3.4.1 Depression

**Figure 3:**
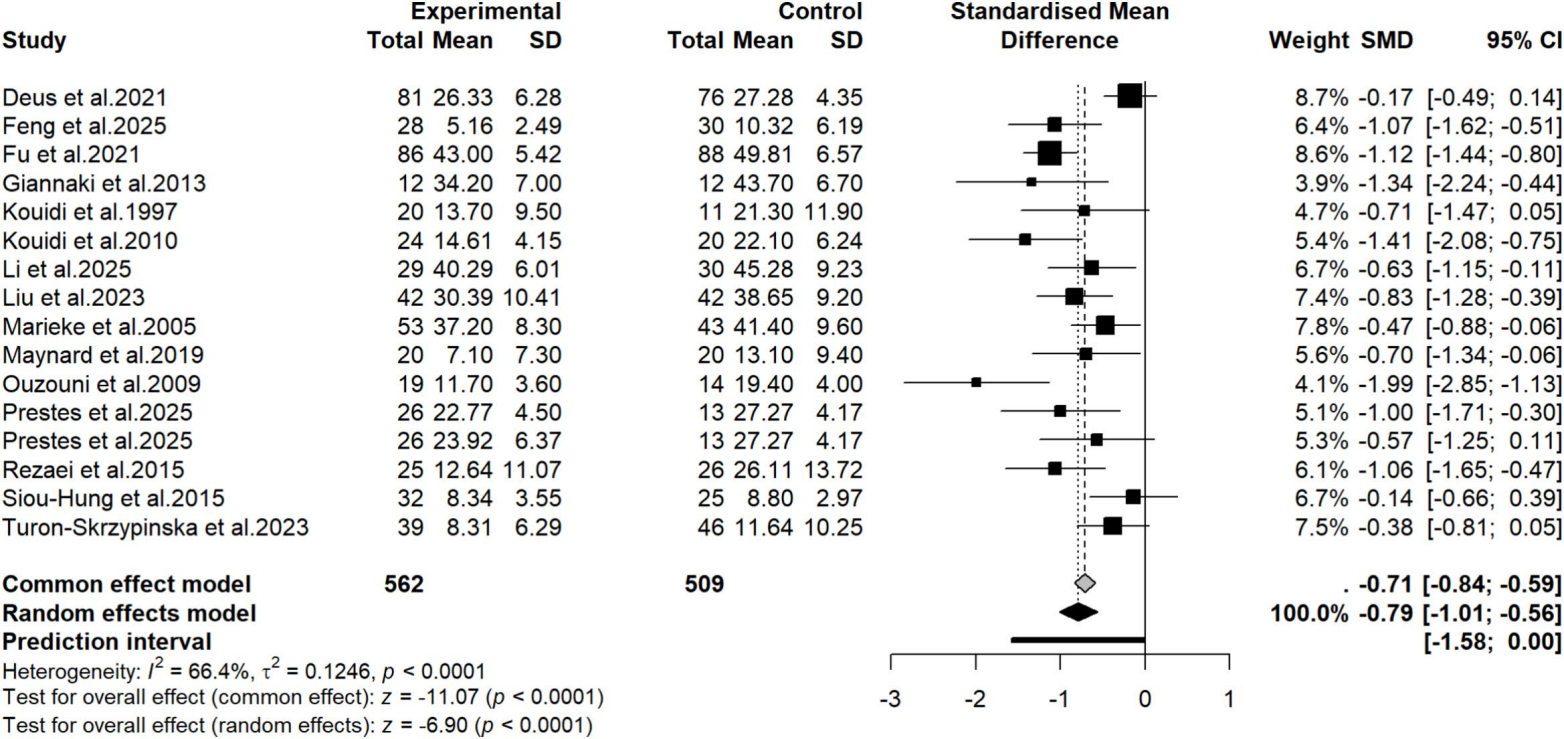
Forest plot of the effectiveness of exercise therapy on depression.

A total of 15 studies (30, 31, 33, 35, 36, 38, 39, 41, 43–46, 51, 54, 55) reported the effect of exercise training on depression. Based on these 15 studies, including 1,071 participants (562 in the intervention group and 509 in the control group) who provided valid data available for pooled analysis, a meta-analysis was performed. Notably, one study (41) included two distinct exercise intervention arms compared against a single control group, resulting in a total of 16 independent cohorts derived from the 15 studies. The pooled results showed that compared with the control group, the depression level of patients significantly decreased after adopting exercise therapy (SMD = −0.79, 95% CI: −1.01 to −0.56, *p* < 0.0001) (Figure 3). The I^2^ statistic and Q test suggested that there was heterogeneity among the pooled studies (I^2^ = 66.4%, *p* < 0.0001). To explore potential sources of heterogeneity, subgroup analysis was performed according to study population characteristics and intervention measures. For the outcome of depression, subgroup analysis were conducted based on age, geographical region, type of exercise, intervention duration, outcome assessment tools, timing of intervention delivery, type of control, the utilization of specialized equipment, exercise load progression, and total weekly exercise volume. Subgroup analysis results showed that in terms of age, the depression improvement effect produced by exercise therapy in patients with an average age under 60 years (SMD = −0.94, 95% CI: −1.13 to −0.74) was significantly better than that in patients with an average age of 60 years and above (SMD = −0.23, 95% CI: −0.45 to 0.00). In terms of exercise type, compared with pure aerobic training (SMD = −0.71, 95% CI: −1.06 to −0.37) or pure strength training (SMD = −0.73, 95% CI: −1.14 to −0.32), combined aerobic and strength training could reduce depressive symptoms to a greater extent (SMD = −0.97, 95% CI: −1.49 to −0.45). In terms of intervention duration, compared with shorter duration exercise (24 weeks or less) (SMD = −0.59, 95% CI: −0.80 to −0.38), longer duration exercise intervention (more than 24 weeks) could reduce depression severity to a greater extent (SMD = −1.23, 95% CI: −1.51 to −0.94). In terms of total weekly exercise volume, compared with an exercise volume of 90 to 120 min (SMD = −0.85, 95% CI: −1.07 to −0.63), exercising for more than 120 min per week could reduce depression severity to the greatest extent (SMD = −0.96, 95% CI: −1.44 to −0.49). However, no statistical difference between groups was seen in the depression improvement effect in subgroups of region, outcome measurement tool, control measure, implementation timing, presence or absence of specialized sports equipment, and exercise load progression. Detailed results of subgroup analysis are shown in Table 3. The meta-regression analysis for the depression outcome showed that age (*p* = 0.002) and intervention duration (*p* = 0.006) significantly moderated the improvement of depression. Other covariates did not show significant associations. Detailed results are presented in Supplementary Table 4.

**Table 3:** Subgroup analysis of the effectiveness of exercise training on depression.

Subgroup	Cohorts	No. of participants (Intervention/Control)	SMD	95% CI	I^2^ (%)	Test of interaction (*p*-value)
Overall	16	562/509	−0.79	−1.01;−0.56	66.4	NA
Region						0.3810
South America	4	153/122	−0.53	−0.92;−0.14	48.9	
East Asia	5	217/215	−0.78	−1.13;−0.44	64.3	
Europe	6	167/146	−0.98	−1.48;−0.47	72.4	
Middle East	1	25/26	−1.06	−1.65;−0.47	NA	
Age						<0.0001
≥60	3	152/147	−0.23	−0.45;0.00	0	
<60	13	410/362	−0.94	−1.13;−0.74	36.2	
Type						0.0282
Strength	5	186/158	−0.73	−1.14;−0.32	70	
Mind–body	1	86/88	−1.12	−1.44;−0.80	NA	
Aerobic	4	113/111	−0.71	−1.06;−0.37	31.3	
Aerobic+Strength	5	145/127	−0.97	−1.49;−0.45	70.8	
Respiratory	1	32/25	−0.14	−0.66;0.39	NA	
Duration						0.0004
24 weeks or less	11	401/364	−0.59	−0.80;−0.38	45.3	
More than 24 weeks	5	161/145	−1.23	−1.51;−0.94	27.8	
Control						0.2782
Education	4	162/132	−0.51	−0.88;−0.15	48.1	
Usual care	10	368/354	−0.86	−1.16;−0.57	68.5	
Sham exercise	1	12/12	−1.34	−2.24;−0.44	NA	
Sedentary	1	20/11	−0.71	−1.47;0.05	NA	
Timing						0.1675
Non-intradialytic	7	296/252	−0.67	−1.01;−0.32	74.9	
Intradialytic	8	213/214	−0.96	−1.29;−0.63	58	
Intradialytic+Non-intradialytic	1	53/43	−0.47	−0.88;−0.06	NA	
Specialized equipment						0.4290
Without	8	333/301	−0.71	−1.01;−0.40	73	
With	8	229/208	−0.89	−1.24;−0.54	61.7	
Scale						0.8068
BDI	9	292/244	−0.76	−1.14;−0.38	73.7	
HAMD	1	28/30	−1.07	−1.62;−0.51	NA	
SDS	5	222/215	−0.84	−1.13;−0.54	50	
CES-D	1	20/20	−0.70	−1.34;−0.06	NA	
Load						0.3397
Progressive	11	327/281	−0.87	−1.16;−0.59	65.1	
Non-progressive	5	235/228	−0.64	−1.02;−0.26	75	
Volume						0.0111
Less than 90 min	2	71/71	−0.28	−0.62;0.05	0	
90 to 120 min	7	283/279	−0.85	−1.07;−0.63	26.4	
More than 120 min	7	208/159	−0.96	−1.44;−0.49	77.4	

BDI, Beck Depression Inventory; CES-D, Center for Epidemiologic Studies Depression Scale; CI, confidence interval; HAMD, Hamilton Depression Rating Scale; NA, Not Applicable; SDS, Self-Rating Depression Scale.

#### 3.4.2 Anxiety

**Figure 4:**
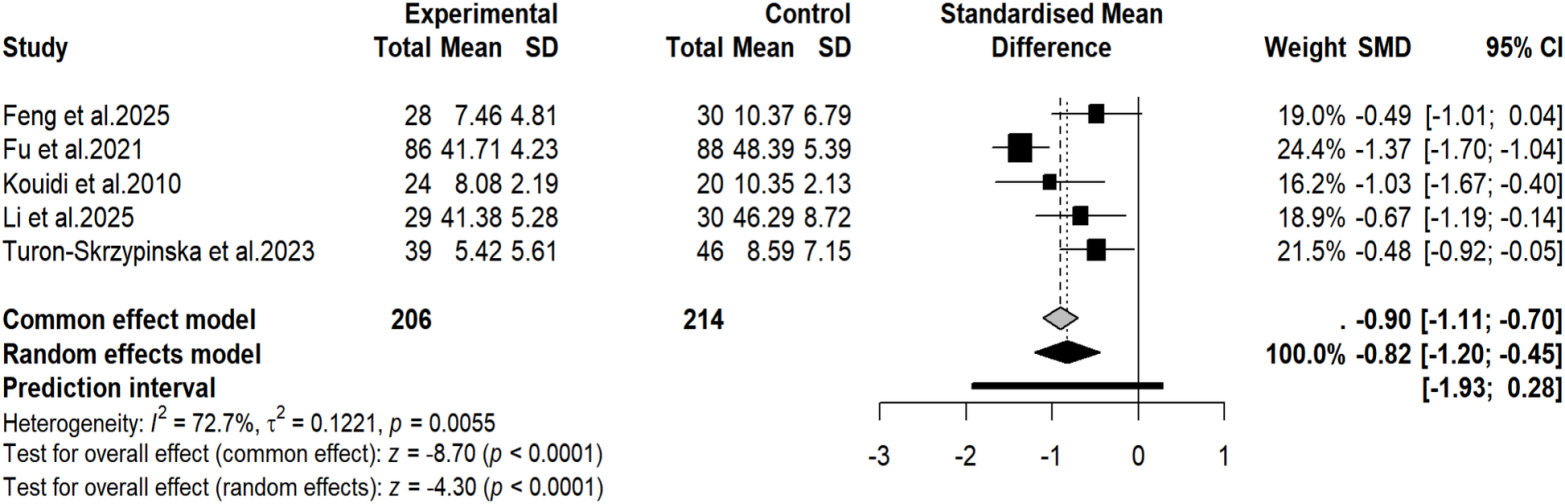
Forest plot of the effectiveness of exercise therapy on anxiety.

A total of 5 studies (30, 31, 33, 45, 55) reported the effect of exercise training on anxiety. Based on these 5 studies, involving 420 participants (206 in the intervention group and 214 in the control group) who provided valid data available for pooled analysis, a meta-analysis was conducted. The pooled results showed that compared with the control group, the anxiety level of patients significantly decreased after adopting exercise therapy (SMD = −0.82, 95% CI: −1.20 to −0.45, *p* < 0.0001) (Figure 4). The I^2^ statistic and Q test suggested that there was heterogeneity among the pooled studies (I^2^ = 72.7%, *p* = 0.0055). Due to the limited number of included studies for this outcome, we only conducted subgroup analysis for intervention duration, region, specialized equipment, and exercise load progression. Other prespecified subgroup domains were not analyzed to avoid unreliable statistical estimates caused by inadequate sample sizes. Subgroup analysis results showed that In terms of intervention duration, compared with shorter duration exercise (24 weeks or less) (SMD = −0.54, 95% CI: −0.82 to −0.26), longer duration exercise intervention (more than 24 weeks) could reduce anxiety severity to a greater extent (SMD = −1.30, 95% CI: −1.59 to −1.00). No statistical difference between groups was seen in the anxiety improvement effect in subgroups of region, presence or absence of specialized sports equipment, and exercise load progression. Detailed results of subgroup analysis are shown in Table 4. The meta-regression analysis for the anxiety outcome revealed that intervention duration (*p* = 0.035) significantly moderated the improvement of anxiety. Other covariates did not show significant associations. Detailed results are presented in Supplementary Table 5. Despite the statistically significant pooled effect size, this finding must be interpreted with extreme caution due to the small number of included studies and substantial statistical heterogeneity.

**Table 4:** Subgroup analysis of the effectiveness of exercise training on anxiety.

Subgroup	Cohorts	No. of participants (Intervention/Control)	SMD	95% CI	I^2^ (%)	Test of interaction (*p*-value)
Overall	5	206/214	−0.82	−1.20;−0.45	72.7	NA
Region						0.6659
East Asia	3	143/148	−0.87	−1.43;−0.32	80.1	
Europe	2	63/66	−0.71	−1.23;−0.18	48.6	
Duration						0.0002
24 weeks or less	3	96/106	−0.54	−0.82;−0.26	0	
More than 24 weeks	2	110/108	−1.30	−1.59;−1.00	0	
Specialized equipment						0.6659
Without	3	143/148	−0.87	−1.43;−0.32	80.1	
With	2	63/66	−0.71	−1.23;−0.18	48.6	
Load						0.6016
Progressive	3	81/80	−0.69	−1.01;−0.37	0	
Non-progressive	2	125/134	−0.94	−1.81;−0.07	90.2	

CI, confidence interval; NA, not applicable.

#### 3.4.3 Physical health-related quality of life

**Figure 5:**
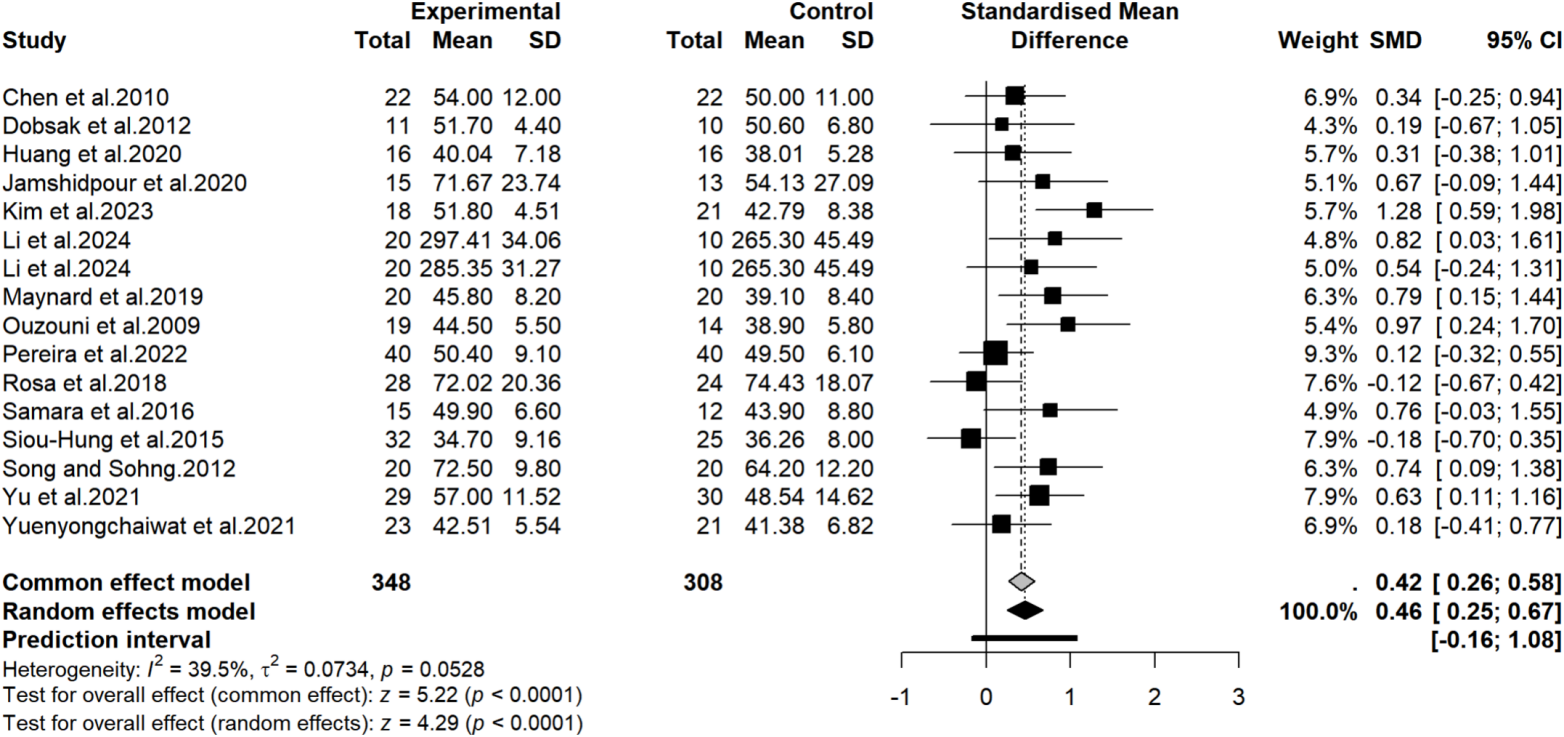
Forest plot of the effectiveness of exercise therapy on physical health-related quality of life.

A total of 15 studies (32, 34, 36, 37, 39, 40, 42, 46–50, 52, 53, 56) reported the effect of exercise training on physical health-related quality of life. Based on these 15 studies, including 656 participants (348 in the intervention group and 308 in the control group) who provided valid data available for pooled analysis, a meta-analysis was performed. One study (22) included two distinct exercise intervention arms compared against a single control group, resulting in a total of 16 independent cohorts derived from the 15 studies. The pooled results showed that compared with the control group, the physical health-related quality of life of patients improved after adopting exercise therapy (SMD = 0.46, 95% CI: 0.25 to 0.67, *p* < 0.0001) (Figure 5). The I^2^ statistic and Q test suggested that there was heterogeneity among the pooled studies (I^2^ = 39.5%, *p* = 0.0528). For the outcome of physical health-related quality of life, subgroup analysis were conducted based on age, geographical region, type of exercise, intervention duration, outcome assessment tools, timing of intervention delivery, type of control, the utilization of specialized equipment, exercise load progression, and total weekly exercise volume. Subgroup analysis results showed that in terms of age, the improvement effect on physical health-related quality of life produced by exercise therapy in patients with an average age under 60 years (SMD = 0.57, 95% CI: 0.33 to 0.81) was significantly better than that in patients with an average age of 60 years and above (SMD = 0.16, 95% CI: −0.12 to 0.44). In terms of specialized sports equipment, patients who used it for exercise training showed greater improvement in physical health-related quality of life (SMD = 0.62, 95% CI: 0.37 to 0.87) than patients who did not use professional equipment or performed bodyweight exercises (SMD = 0.20, 95% CI: −0.09 to 0.49). However, no statistical difference between groups was seen in the physical health-related quality of life improvement effect in subgroups of region, exercise type, intervention duration, outcome measurement tool, control measure, implementation timing, exercise load progression, and total weekly exercise volume. Detailed results of subgroup analysis are shown in Table 5. The meta-regression analysis for the physical health-related quality of life outcome demonstrated that none of the covariates exhibited a significant moderating effect on the intervention outcomes. Detailed results are presented in Supplementary Table 6.

**Table 5:** Subgroup analysis of the effectiveness of exercise training on physical health-related quality of life.

Subgroup	Cohorts	No. of participants (Intervention/Control)	SMD	95% CI	I^2^ (%)	Test of interaction (*p*-value)
Overall	16	348/308	0.46	0.25;0.67	39.5	NA
Region						0.6401
North America	1	22/22	0.34	−0.25;0.94	NA	
Europe	3	45/36	0.68	0.22;1.14	0	
East Asia	7	155/132	0.56	0.20;0.92	53.5	
Middle East	1	15/13	0.67	−0.09;1.44	NA	
South America	3	88/84	0.23	−0.26;0.71	57.4	
Southeast Asia	1	23/21	0.18	−0.41;0.77	NA	
Age						0.0283
≥60	4	109/100	0.16	−0.12;0.44	18.9	
<60	12	239/208	0.57	0.33;0.81	29.7	
Type						0.1064
Strength	3	70/66	0.29	−0.19;0.78	50.7	
Aerobic	5	104/93	0.60	0.14;1.07	57.3	
Aerobic+Strength	5	99/93	0.67	0.38;0.96	0	
Respiratory	3	75/56	0.10	−0.28;0.49	15.3	
Duration						0.1738
24 weeks or less	14	300/264	0.41	0.19;0.64	39.9	
More than 24 weeks	2	48/44	0.75	0.32;1.17	0	
Control						0.1929
Sham exercise	4	89/83	0.15	−0.15;0.45	0	
Usual care	9	186/152	0.55	0.28;0.82	26	
Education	2	58/61	0.67	−0.48;1.81	87.1	
Sedentary	1	15/12	0.76	−0.03;1.55	NA	
Timing						0.8185
Non-intradialytic	3	67/57	0.40	−0.24;1.04	68	
Intradialytic	13	281/251	0.48	0.25;0.70	33.6	
Specialized equipment						0.0333
Without	6	145/122	0.20	−0.09;0.49	27.8	
With	10	203/186	0.62	0.37;0.87	23.3	
Scale						0.0747
SF-36	13	269/231	0.54	0.29;0.79	44	
SF-12	3	79/77	0.17	−0.14;0.49	0	
Load						0.7984
Progressive	12	247/233	0.48	0.24; 0.72	39.1	
Non-progressive	4	101/75	0.41	−0.06;0.88	54.4	
Volume						0.0905
Less than 90 min	4	92/81	0.19	−0.14;0.53	17.8	
90 to 120 min	9	193/171	0.49	0.18;0.79	47.2	
More than 120 min	3	63/56	0.75	0.37;1.13	0	

CI, confidence interval; NA, not applicable; SF-12, Short Form 12 Health Survey; SF-36, Short Form 36 Health Survey.

### 3.5 Sensitivity analysis

Sensitivity analyses were conducted for depression, anxiety, and physical health-related quality of life using the leave-one-out method, in which individual studies were sequentially excluded. For depression, the sequential exclusion of any single study did not substantially alter the pooled effect size or heterogeneity, indicating that the overall findings are robust. For anxiety, the removal of the study by Fu et al. (31) significantly reduced heterogeneity (from I^2^ = 72.7 to 0%). Even after excluding this study, the synthesized result continued to support the efficacy of exercise therapy (SMD = −0.62, 95% CI: −0.88 to −0.36, *p* < 0.0001). For physical health-related quality of life, similar to depression, the pooled results and heterogeneity statistics exhibited minimal fluctuation upon the exclusion of any individual study, underscoring the stability of the overall synthesized effect. Detailed results of the sensitivity analyses are available in Supplementary Figures 2–4.

### 3.6 Publication bias

We used Egger’s test and funnel plot analysis to assess publication bias for depression, anxiety, and physical health-related quality of life. Because judging whether the funnel plot is symmetrical with the naked eye has subjectivity, we also performed Egger’s test and used it as the basis for judging publication bias. The results showed that there was no publication bias for the outcomes of depression (*p* = 0.0840) and anxiety (*p* = 0.2879), but there was potential publication bias for the physical health-related quality of life outcome (*p* = 0.0220). We further carried out trim-and-fill method analysis. The results showed that the improvement effect of exercise on depression (SMD = −0.6633, 95% CI: −0.9113 to −0.4153, *p* = < 0.0001), anxiety (SMD = −1.1022, 95% CI: −1.5376 to −0.6668, *p* < 0.0001), and physical health-related quality of life (SMD = 0.2787, 95% CI: 0.0556 to 0.5017, *p* = 0.0143) was still statistically significant. However, for the anxiety outcome, because only 5 studies were included, the statistical reliability of both Egger’s test and the trim-and-fill analysis is highly limited. Therefore, these results should be interpreted with extreme caution, and the possibility of publication bias cannot be completely ruled out. Detailed results of the publication bias assessments are provided in Supplementary Figures 5–10.

## 4 Discussion

Maintenance hemodialysis patients often experience a substantial psychological burden. Depression and anxiety are common complications in this population, which not only severely impair their quality of life but also increase the risk of adverse clinical outcomes. Therefore it is of high clinical urgency to identify effective and safe interventions to alleviate these negative emotions. This systematic review and meta-analysis of randomized controlled trials demonstrates that exercise training can significantly improve depressive symptoms in patients undergoing maintenance hemodialysis, while modestly enhancing their physical health-related quality of life. Regarding the anxiety outcome, the findings must be interpreted with extreme caution. Although the pooled analysis revealed a statistically significant reduction in anxiety severity, this result is derived from a limited evidence base of only five studies. Furthermore, the substantial heterogeneity among these studies, combined with the methodological limitation of being unable to reliably assess publication bias due to the small sample size, severely weakens the certainty of this finding. Therefore, at this stage, this study considers the ameliorating effect of exercise training on anxiety to be a purely exploratory analysis rather than a definitive conclusion.

According to previous literature, the process by which exercise therapy improves the mental state of MHD patients may involve an integrated mechanism. Biologically, long-term MHD patients are frequently in a chronic uremic proinflammatory environment, and high circulating levels of IL-6 and TNF-*α* may be associated with the pathogenesis of depression (57, 58). However, regular exercise can induce skeletal muscles to secrete myokines to exert anti-inflammatory effects, which might play a role in emotional regulation (59). On the other hand, exercise training may be associated with the significant expression of brain-derived neurotrophic factor (BDNF) (60, 61). As a key regulator of neuroplasticity, BDNF can repair hippocampal damage and synaptic atrophy caused by uremic toxins (62, 63). This structural repair might help counteract negative emotions. Regular exercise can also cyclically regulate cortisol levels (64), which may reduce patients’ excessive responses to stressors to some extent. Psychologically, due to the absolute dependence on dialysis machines, forced changes in life rhythm, and multiple complications, MHD patients may develop learned helplessness (65), a psychological state that frequently triggers negative emotions. However, the positive feedback gained from participating in exercise might alleviate some of this helplessness (66). During training, the brain must process body balance, muscle strength exertion, and breathing rhythm, a diversion of attention that may reduce patients’ focus on the disease (67). Additionally, bedside or dialysis room exercise increases interpersonal social interactions to a certain extent (41). This informal social support network might also exert a positive impact.

In our study, Egger’s test indicated potential publication bias regarding physical health-related quality of life (*p* = 0.0220). After adjustment using the trim-and-fill method, SMD decreased from 0.46 to 0.2787. Although the adjusted effect size remained statistically significant (*p* = 0.0143), an SMD of 0.2787 constitutes a small effect size statistically. This attenuation of the effect size suggests that the initial pooled effect estimate may have been partially overestimated due to the nonpublication of studies with negative or nonstatistically significant results. MHD patients are chronically exposed to a uremic microenvironment and frequently present with chronic inflammation, protein energy wasting and severe fatigue related to dialysis. These factors severely blunt the physiological translation of generic exercise stimuli into robust improvements in physical functioning. This also suggests that healthcare professionals should appropriately contextualize the clinical benefits of exercise. Exercise training alone may yield only minor physical improvements for MHD patients. Therefore, combined protocols should be actively explored. For instance, while developing high quality exercise programs for patients, healthcare professionals could simultaneously implement measures such as individualized nutritional interventions. This combined approach might provide a more effective enhancement of physical health-related quality of life for this patient population. Furthermore, the present study found that the included trials generally lacked detailed reporting on blinding procedures. When evaluating subjective self-reported outcomes, the absence of blinding is highly likely to introduce significant performance bias and detection bias. Because of the specific nature of exercise interventions, patients aware of their allocation to the exercise group might experience a strong placebo effect. This awareness could lead them to report more positive psychological and physiological states on subjective scales. Additionally, unblinded outcome assessors might inadvertently guide patients toward responses favoring the efficacy of the intervention. These factors have a high probability of partially inflating the observed effect sizes. Consequently, we downgraded the certainty of evidence during the GRADE assessment to account for this limitation. Future research must rigorously implement and detail blinded outcome assessment mechanisms to obtain more objective and accurate effect estimates.

A key finding of this study is that there may be a benefit bottleneck for exercise intervention in older MHD patients aged 60 and above. Whether it is relieving psychological symptoms, or improving physical health-related quality of life, their effect sizes are lower than patients under 60. This difference in physical and mental benefits reveals that older patients may have multi-dimensional biological and psychological barriers when coping with exercise stimulation. The Clinical Practice Guideline for the Evaluation and Management of Chronic Kidney Disease published by the 2024 KDIGO CKD Work Group also points out that patient age is an important consideration for implementing exercise programs (68). For this, a possible explanation is that with advancing age, the sensitivity of the central nervous system to exercise stimulation decreases (69), meaning that a higher exercise threshold is required to produce the same beneficial effects on negative emotions. However, older MHD patients are generally accompanied by severe uremic sarcopenia and protein-energy wasting (70), meaning the exercise intensity they can tolerate often fails to reach the effective dose for neuronal repair. Older MHD patients are also a high-incidence group for cognitive impairment (71), which directly affects their understanding and execution of complex exercise prescriptions. In the absence of high-intensity supervision, older adults are prone to improper exercise techniques, and participants may reduce exercise intensity or frequency below the prescribed level, which may lead to a smaller observed effect size. From the perspective of clinical psychology in dialysis patients, older MHD patients generally face higher mortality risk and the loss of social role functions (72), which are further exacerbated by their absolute dependence on dialysis machines and strict treatment schedules. This entrenched psychological distress may make it difficult for simple exercise therapy to substantially improve their psychological symptoms. Regarding the physical health dimension, older patients are often accompanied by anabolic resistance (73). Under the constant chronic inflammation and amino acid loss inherent to the hemodialysis environment, their muscle protein synthesis efficiency is significantly lower than that of younger patients. This suggests that even if they complete the same frequency of exercise, older patients may find it difficult to translate this effort into effective gains in muscle mass and physical fitness. And, because older patients have less overall physiological reserve, their physical function has been limited to a lower level. In addition, compared with young people, older patients have a long physiological repair cycle after exercise intervention. The unique post-dialysis fatigue caused by hemodynamic shifts and ultrafiltration, combined with exercise load, may overwhelm their limited physiological reserve. This sense of fatigue may offset the positive subjective feeling brought by weak physical progress, leading to insignificant final effect size. However, these possible explanations are not intended to dismiss the value of exercise therapy, but rather to indicate that simple exercise therapy alone may be insufficient for older MHD patients. Another finding of this study is that the effect of using specialized equipment, such as bedside cycle ergometers and virtual reality devices, on improving physical health-related quality of life was better than simple equipment, such as elastic bands or bare-handed exercise, but showed no significant advantage in depression and anxiety outcomes. It is speculated that the reason may be that specialized equipment allows healthcare professionals to precisely adjust the load with very small increments (74), which is conducive to patients always exercising within the effective stimulation threshold. In contrast, simple equipment or bodyweight exercise is often difficult to maintain constant and increasing load, leading to a plateau in physical function benefits. The physical support and ergonomic kinetic path provided by specialized equipment can not only effectively stimulate the training site but also reduce the risk of sports injury caused by posture changes or improper force exertion (75), making patients complete the exercise plan more efficiently and safely. We also observed that interventions exceeding 24 weeks and adopting combined aerobic and strength training with a total weekly exercise volume of more than 120 min were more effective for the relief of negative emotions. This may be because the improvement of psychological conditions in MHD patients is a long-term adaptation process. Given their rigid dialysis schedules and chronic fatigue, patients require an extended period to overcome initial exercise intolerance and gradually turn exercise into a sustainable lifestyle, an effect that short-term interventions cannot achieve. Furthermore, this combined modality addresses the specific clinical needs of the dialysis population. Previous literature suggests that aerobic exercise may induce the release of endorphins and dopamine to produce immediate psychological relief. Meanwhile, strength training is particularly crucial for counteracting uremic sarcopenia and dialysis-induced muscle wasting. The combination of these two modalities produces a clinical synergistic effect, comprehensively addressing both the physiological and psychological burdens of MHD patients. It is worth noting that only one included study (Fu et al.) explored mind–body exercise rooted in Traditional Chinese Medicine Qigong. While this low-impact, equipment-free modality may offer a potential alternative for MHD patients intolerant to high-intensity regimens, it represents an isolated finding in our review. Therefore, firm conclusions regarding the overall efficacy of mind–body exercise cannot be drawn at this stage. Future large-scale randomized controlled trials are warranted to rigorously evaluate its specific impact on clinical outcomes. The control conditions in the included studies exhibited clinical heterogeneity, primarily comprising usual care, sham exercise, health education, and sedentary behavior. We pooled these conditions to evaluate the overall efficacy of exercise interventions relative to a state lacking substantial exercise stimuli. Our subgroup analyses showed that the differences in effect sizes among the various control groups were not statistically significant. This supports the feasibility of the pooled analysis at the data level and indicates that the positive effects of exercise training remain relatively consistent regardless of the basic care intervention received by the control group. However, these control conditions theoretically differ in their ability to control for placebo effects. Therefore, we recommend that future clinical trials adopt more standardized control designs.

Previous similar reviews had a relatively single focus, only including intradialytic exercise (76, 77) or only including aerobic exercise (78), and the outcomes mainly focused on physical improvements, without deeply discussing psychological outcomes, conducting detailed subgroup analysis, and exploring sources of heterogeneity. In contrast, the present study incorporated a wider variety of exercise modalities, conducted more comprehensive subgroup analyses, performed meta-regression and sensitivity analysis, and placed greater emphasis on addressing the research gap regarding psychological outcomes in MHD patients. With the increasing emphasis on precision care and the continuous emergence of new evidence, a single exercise type may no longer suffice to meet the diverse needs of MHD patients. This necessitates that healthcare professionals promptly develop targeted, personalized exercise plans and explore the efficacy of exercise therapy across multiple dimensions. This study provided relatively precise evidence on subdivided dimensions, explicitly pointing out that age, exercise type, duration, and presence or absence of specialized equipment may be important variables affecting clinical outcomes, which may also explain why there are large gaps between some research results. Based on the findings of this study, we recommend that healthcare professionals prioritize a combined aerobic and strength training modality when implementing exercise programs for MHD patients. The intervention duration should exceed 24 weeks with a total weekly exercise volume of ideally more than 120 min. Additionally, specialized equipment should be utilized whenever feasible. For MHD patients aged 60 and older, clinicians should simultaneously consider incorporating other auxiliary interventions while developing individualized exercise training protocols.

This study also has some limitations. First, due to the inconsistent reporting and absence of data regarding the baseline severity of depression and anxiety, dialysis vintage and comorbidity burden among the included studies, we did not conduct an in-depth analysis of these variables. This limitation may have affected our exploration of heterogeneity in the present study. Second, the evidence base for the anxiety outcome is highly limited, encompassing only five studies. Such a small sample size not only limits the statistical power and generalizability of the conclusions but also severely restricts the reliable assessment of publication bias and the exploration of sources driving the high heterogeneity. Therefore, the ameliorating effect of exercise training on anxiety cannot be definitively confirmed. Third, potential publication bias was detected in the physical health-related quality of life outcome. Although trim-and-fill adjustment confirms the continued positive effect of exercise interventions, the noticeably reduced effect size indicates that the current pooled results may overestimate the true therapeutic benefit. Finally, most studies failed to report on the blinding of outcome assessors. The potential lack of blinding might influence the observed effect sizes and consequently reduce the overall certainty of the evidence. However, although this study has some limitations, it can still provide some valuable insights.

## 5 Conclusion

In conclusion, this systematic review and meta-analysis of 27 randomized controlled trials demonstrates that exercise interventions effectively ameliorate depression and modestly enhance physical health-related quality of life in patients receiving maintenance hemodialysis. However, due to the limited evidence base of only five studies, high statistical heterogeneity, and restricted assessment of publication bias, the therapeutic effect of exercise on anxiety remains uncertain. These exploratory findings require future large-scale and rigorously designed randomized controlled trials for further verification. Crucially, we identified the roles of age, equipment utilization, exercise type, intervention duration, and total weekly exercise volume in moderating the intervention effects. Future research should prioritize the development of personalized exercise prescriptions for hemodialysis patients, while concurrently enhancing the methodological rigor of study designs. Clinically, Healthcare professionals should also pay more attention to improving the clinical outcomes of this vulnerable group by implementing exercise interventions.

## Data Availability

The original contributions presented in the study are included in the article/Supplementary material, further inquiries can be directed to the corresponding author.
