# Supplementary material for: Effects of exercise training on depression, anxiety, and physical health-related quality of life in end-stage renal disease patients receiving maintenance hemodialysis: a systematic review and meta-analysis of randomized controlled trials
Source: Front Public Health. 2026 Apr 23;14:1788845. doi: 10.3389/fpubh.2026.1788845 (PMC13149201; doi:10.3389/fpubh.2026.1788845)
Supplement: Supplementary file 1 [file Supplementary_file_1.docx]

***Supplementary Material***

**Supplementary Table 1.** Search strategy

| ****PubMed 473 Results**** | |
| --- | --- |
| **#1** | ((((((((("Renal Dialysis"[Mesh]) OR (Dialyses, Renal[Title/Abstract])) OR (Renal Dialyses[Title/Abstract])) OR (Dialysis, Renal[Title/Abstract])) OR (Hemodialysis[Title/Abstract])) OR (Hemodialyses[Title/Abstract])) OR (Dialysis, Extracorporeal[Title/Abstract])) OR (Dialyses, Extracorporeal[Title/Abstract])) OR (Extracorporeal Dialyses[Title/Abstract])) OR (Extracorporeal Dialysis[Title/Abstract]) |
| **#2** | ((((((((((((((((((((((((("Exercise"[Mesh]) OR (Exercises[Title/Abstract])) OR (Exercise, Physical[Title/Abstract])) OR (Exercises, Physical[Title/Abstract])) OR (Physical Exercise[Title/Abstract])) OR (Physical Exercises[Title/Abstract])) OR (Exercise, Aerobic[Title/Abstract])) OR (Aerobic Exercise[Title/Abstract])) OR (Aerobic Exercises[Title/Abstract])) OR (Exercises, Aerobic[Title/Abstract])) OR (Exercise, Isometric[Title/Abstract])) OR (Exercises, Isometric[Title/Abstract])) OR (Isometric Exercises[Title/Abstract])) OR (Isometric Exercise[Title/Abstract])) OR (Acute Exercise[Title/Abstract])) OR (Acute Exercises[Title/Abstract])) OR (Exercise, Acute[Title/Abstract])) OR (Exercises, Acute[Title/Abstract])) OR (Exercise Training[Title/Abstract])) OR (Exercise Trainings[Title/Abstract])) OR (Training, Exercise[Title/Abstract])) OR (Trainings, Exercise[Title/Abstract])) OR (Physical Activity[Title/Abstract])) OR (Activities, Physical[Title/Abstract])) OR (Activity, Physical[Title/Abstract])) OR (Physical Activities[Title/Abstract]) |
| **#3** | ((((((("Depression"[Mesh]) OR (Depressive Symptoms[Title/Abstract])) OR (Depressive Symptom[Title/Abstract])) OR (Symptom, Depressive[Title/Abstract])) OR (Emotional Depression[Title/Abstract])) OR (Depression, Emotional[Title/Abstract])) OR ((((((((("Anxiety"[Mesh]) OR (Angst[Title/Abstract])) OR (Nervousness[Title/Abstract])) OR (Hypervigilance[Title/Abstract])) OR (Social Anxiety[Title/Abstract])) OR (Anxieties, Social[Title/Abstract])) OR (Anxiety, Social[Title/Abstract])) OR (Social Anxieties[Title/Abstract])) OR (Anxiousness[Title/Abstract]))) OR ((((("Quality of Life"[Mesh]) OR (Life Quality[Title/Abstract])) OR (Health-Related Quality Of Life[Title/Abstract])) OR (Health Related Quality Of Life[Title/Abstract])) OR (HRQOL[Title/Abstract])) |
| **#4** | #1 AND #2 AND #3 |
| ****Web of Science 1403 Results**** | |
| **#1** | Renal Dialysis (Topic) or Dialyses, Renal (Topic) or Renal Dialyses (Topic) or Dialysis, Renal (Topic) or Hemodialysis (Topic) or Hemodialyses (Topic) or Dialysis, Extracorporeal (Topic) or Dialyses, Extracorporeal (Topic) or Extracorporeal Dialyses (Topic) or Extracorporeal Dialysis (Topic) |
| **#2** | Exercise (Topic) or Exercises (Topic) or Exercise, Physical (Topic) or Exercises, Physical (Topic) or Physical Exercise (Topic) or Physical Exercises (Topic) or Exercise, Aerobic (Topic) or Aerobic Exercise (Topic) or Aerobic Exercises (Topic) or Exercises, Aerobic (Topic) or Exercise, Isometric (Topic) or Exercises, Isometric (Topic) or Isometric Exercises (Topic) or Isometric Exercise (Topic) or Acute Exercise (Topic) or Acute Exercises (Topic) or Exercise, Acute (Topic) or Exercises, Acute (Topic) or Exercise Training (Topic) or Exercise Trainings (Topic) or Training, Exercise (Topic) or Trainings, Exercise (Topic) or Physical Activity (Topic) or Activities, Physical (Topic) or Activity, Physical (Topic) or Physical Activities (Topic) |
| **#3** | Depression (Topic) or Depressive Symptoms (Topic) or Depressive Symptom (Topic) or Symptom, Depressive (Topic) or Emotional Depression (Topic) or Depression, Emotional (Topic) or Anxiety (Topic) or Angst (Topic) or Nervousness (Topic) or Hypervigilance (Topic) or Social Anxiety (Topic) or Anxieties, Social (Topic) or Anxiety, Social (Topic) or Social Anxieties (Topic) or Anxiousness (Topic) or Quality of Life (Topic) or Life Quality (Topic) or Health-Related Quality Of Life (Topic) or Health Related Quality Of Life (Topic) or HRQOL (Topic) |
| **#4** | #1 AND #2 AND #3 |
| ****CENTRAL 580 Results**** | |
| **#1** | MeSH descriptor: [Renal Dialysis] explode all trees |
| **#2** | (Dialyses, Renal):ti,ab,kw OR (Renal Dialyses):ti,ab,kw OR (Dialysis, Renal):ti,ab,kw OR (Hemodialysis):ti,ab,kw OR (Hemodialyses):ti,ab,kw OR (Dialysis, Extracorporeal):ti,ab,kw OR (Dialyses, Extracorporeal):ti,ab,kw OR (Extracorporeal Dialyses):ti,ab,kw OR (Extracorporeal Dialysis):ti,ab,kw |
| **#3** | **#1 OR #2** |
| **#4** | MeSH descriptor: [Exercise] explode all trees |
| **#5** | (Exercises):ti,ab,kw OR (Exercise, Physical):ti,ab,kw OR (Exercises, Physical):ti,ab,kw OR (Physical Exercise):ti,ab,kw OR (Physical Exercises):ti,ab,kw OR (Exercise, Aerobic):ti,ab,kw OR (Aerobic Exercise):ti,ab,kw OR (Aerobic Exercises):ti,ab,kw OR (Exercises, Aerobic):ti,ab,kw OR (Exercise, Isometric):ti,ab,kw OR (Exercises, Isometric):ti,ab,kw OR (Isometric Exercises):ti,ab,kw OR (Isometric Exercise):ti,ab,kw OR (Acute Exercise):ti,ab,kw OR (Acute Exercises):ti,ab,kw OR (Exercise, Acute):ti,ab,kw OR (Exercises, Acute):ti,ab,kw OR (Exercise Training):ti,ab,kw OR (Exercise Trainings):ti,ab,kw OR (Training, Exercise):ti,ab,kw OR (Trainings, Exercise):ti,ab,kw OR (Physical Activity):ti,ab,kw OR (Activities, Physical):ti,ab,kw OR (Activity, Physical):ti,ab,kw OR (Physical Activities):ti,ab,kw |
| **#6** | **#4 OR #5** |
| **#7** | MeSH descriptor: [Depression] explode all trees |
| **#8** | (Depressive Symptoms):ti,ab,kw OR (Depressive Symptom):ti,ab,kw OR (Symptom, Depressive):ti,ab,kw OR (Emotional Depression):ti,ab,kw OR (Depression, Emotional):ti,ab,kw |
| **#9** | MeSH descriptor: [Anxiety] explode all trees |
| **#10** | (Angst):ti,ab,kw OR (Nervousness):ti,ab,kw OR (Hypervigilance):ti,ab,kw OR (Social Anxiety):ti,ab,kw OR (Anxieties, Social):ti,ab,kw OR (Anxiety, Social):ti,ab,kw OR (Social Anxieties):ti,ab,kw OR (Anxiousness):ti,ab,kw |
| **#11** | MeSH descriptor: [Quality of Life] explode all trees |
| **#12** | (Life Quality):ti,ab,kw OR (Health-Related Quality Of Life):ti,ab,kw OR (Health Related Quality Of Life):ti,ab,kw OR (HRQOL):ti,ab,kw |
| **#13** | **#7 OR #8 OR #9 OR #10 OR #11 OR #12** |
| **#14** | **#3 AND #6 AND #13** |
| ****Embase 1422 Results**** | |
| **#1** | 'hemodialysis'/exp |
| **#2** | 'renal dialysis':ab,kw,ti OR 'dialyses, renal':ab,kw,ti OR 'renal dialyses':ab,kw,ti OR 'dialysis, renal':ab,kw,ti OR 'hemodialysis':ab,kw,ti OR 'hemodialyses':ab,kw,ti OR 'dialysis, extracorporeal':ab,kw,ti OR 'dialyses, extracorporeal':ab,kw,ti OR 'extracorporeal dialyses':ab,kw,ti OR 'extracorporeal dialysis':ab,kw,ti |
| **#3** | **#1 OR #2** |
| **#4** | **'exercise'/exp** |
| **#5** | **'exercise':ab,kw,ti OR 'exercises':ab,kw,ti OR 'exercise, physical':ab,kw,ti OR 'exercises, physical':ab,kw,ti OR 'physical exercise':ab,kw,ti OR 'physical exercises':ab,kw,ti OR 'exercise, aerobic':ab,kw,ti OR 'aerobic exercise':ab,kw,ti OR 'aerobic exercises':ab,kw,ti OR 'exercises, aerobic':ab,kw,ti OR 'exercise, isometric':ab,kw,ti OR 'exercises, isometric':ab,kw,ti OR 'isometric exercises':ab,kw,ti OR 'isometric exercise':ab,kw,ti OR 'acute exercise':ab,kw,ti OR 'acute exercises':ab,kw,ti OR 'exercise, acute':ab,kw,ti OR 'exercises, acute':ab,kw,ti OR 'exercise training':ab,kw,ti OR 'exercise trainings':ab,kw,ti OR 'training, exercise':ab,kw,ti OR 'trainings, exercise':ab,kw,ti OR 'physical activity':ab,kw,ti OR 'activities, physical':ab,kw,ti OR 'activity, physical':ab,kw,ti OR 'physical activities':ab,kw,ti** |
| **#6** | **#4 OR #5** |
| **#7** | **'depression'/exp** |
| **#8** | **'depression':ab,kw,ti OR 'depressive symptoms':ab,kw,ti OR 'depressive symptom':ab,kw,ti OR 'symptom, depressive':ab,kw,ti OR 'emotional depression':ab,kw,ti OR 'depression, emotional':ab,kw,ti** |
| **#9** | **'anxiety'/exp** |
| **#10** | **'anxiety':ab,kw,ti OR 'angst':ab,kw,ti OR 'nervousness':ab,kw,ti OR 'hypervigilance':ab,kw,ti OR 'social anxiety':ab,kw,ti OR 'anxieties, social':ab,kw,ti OR 'anxiety, social':ab,kw,ti OR 'social anxieties':ab,kw,ti OR 'anxiousness':ab,kw,ti** |
| **#11** | **'quality of life'/exp** |
| **#12** | **'quality of life':ab,kw,ti OR 'life quality':ab,kw,ti OR 'health-related quality of life':ab,kw,ti OR 'health related quality of life':ab,kw,ti OR 'hrqol':ab,kw,ti** |
| **#13** | **#7 OR #8 OR #9 OR #10 OR #11 OR #12** |
| **#14** | **#3 AND #6 AND #13** |
| ****MEDLINE 615 Results**** | |
| **#1** | **Renal Dialysis/** |
| **#2** | **(Renal Dialysis or renal dialysis or dialyses, renal or renal dialyses or dialysis, renal or hemodialysis or hemodialyses or dialysis, extracorporeal or dialyses, extracorporeal or extracorporeal dialyses or extracorporeal dialysis).ab,ti,kw.** |
| **#3** | **#1 OR #2** |
| **#4** | **Exercise/** |
| **#5** | **(Exercise or exercise or exercises or exercise, physical or exercises, physical or physicalphysical exercises or exercise, aerobic or aerobicaerobic exercises or exercises, aerobic or exercise, isometric or exercises, isometric or isometric exercises or isometricacuteacute exercises or exercise, acute or exercises, acute or exercise training or exercise trainings or training,trainings,physical activity or activities, physical or activity, physical or physical activities).ab,ti,kw.** |
| **#6** | **#4 OR #5** |
| **#7** | **Depression/** |
| **#8** | **(Depression or depression or depressive symptoms or depressive symptom or symptom, depressive or emotionaldepression, emotional).ab,ti,kw.** |
| **#9** | **Anxiety/** |
| **#10** | **(Anxiety or anxiety or angst or nervousness or hypervigilance or socialanxieties, social or anxiety, social or social anxieties or anxiousness).ab,ti,kw.** |
| **#11** | **"Quality of Life"/** |
| **#12** | **(quality of life or life quality or health-relatedhealth relatedhrqol).ab,ti,kw.** |
| **#13** | **#7 OR #8 OR #9 OR #10 OR #11 OR #12** |
| **#14** | **#3 AND #6 AND #13** |
| ****Scopus 1494 Results**** | |
| **#1** | TITLE-ABS-KEY ( "Renal Dialysis" OR "Dialyses, Renal" OR "Renal Dialyses" OR "Dialysis, Renal" OR "Hemodialysis" OR "Hemodialyses" OR "Dialysis, Extracorporeal" OR "Dialyses, Extracorporeal" OR "Extracorporeal Dialyses" OR "Extracorporeal Dialysis" ) |
| **#2** | TITLE-ABS-KEY ( "Exercise" OR "Exercises" OR "Exercise, Physical" OR "Exercises, Physical" OR "Physical Exercise" OR "Physical Exercises" OR "Exercise, Aerobic" OR "Aerobic Exercise" OR "Aerobic Exercises" OR "Exercises, Aerobic" OR "Exercise, Isometric" OR "Exercises, Isometric" OR "Isometric Exercises" OR "Isometric Exercise" OR "Acute Exercise" OR "Acute Exercises" OR "Exercise, Acute" OR "Exercises, Acute" OR "Exercise Training" OR "Exercise Trainings" OR "Training, Exercise" OR "Trainings, Exercise" OR "Physical Activity" OR "Activities, Physical" OR "Activity, Physical" OR "Physical Activities" ) |
| **#3** | TITLE-ABS-KEY ( "Depression" OR "Depressive Symptoms" OR "Depressive Symptom" OR "Symptom, Depressive" OR "Emotional Depression" OR "Depression, Emotional" OR "Anxiety" OR "Angst" OR "Nervousness" OR "Hypervigilance" OR "Social Anxiety" OR "Anxieties, Social" OR "Anxiety, Social" OR "Social Anxieties" OR "Anxiousness" OR "Quality of Life" OR "Life Quality" OR "Health-Related Quality Of Life" OR "Health Related Quality Of Life" OR "HRQOL"  ) |
| **#4** | #1 AND #2 AND #3 |
| ****CINAHL 239 Results**** | |
| **#1** | SU (Renal Dialysis) OR TI ("Renal Dialysis" OR "Dialyses, Renal" OR "Renal Dialyses" OR "Dialysis, Renal" OR "Hemodialysis" OR "Hemodialyses" OR "Dialysis, Extracorporeal" OR "Dialyses, Extracorporeal" OR "Extracorporeal Dialyses" OR "Extracorporeal Dialysis") OR AB ("Renal Dialysis" OR "Dialyses, Renal" OR "Renal Dialyses" OR "Dialysis, Renal" OR "Hemodialysis" OR "Hemodialyses" OR "Dialysis, Extracorporeal" OR "Dialyses, Extracorporeal" OR "Extracorporeal Dialyses" OR "Extracorporeal Dialysis") |
| **#2** | SU (Exercise) OR TI ("Exercise" OR "Exercises" OR "Exercise, Physical" OR "Exercises, Physical" OR "Physical Exercise" OR "Physical Exercises" OR "Exercise, Aerobic" OR "Aerobic Exercise" OR "Aerobic Exercises" OR "Exercises, Aerobic" OR "Exercise, Isometric" OR "Exercises, Isometric" OR "Isometric Exercises" OR "Isometric Exercise" OR "Acute Exercise" OR "Acute Exercises" OR "Exercise, Acute" OR "Exercises, Acute" OR "Exercise Training" OR "Exercise Trainings" OR "Training, Exercise" OR "Trainings, Exercise" OR "Physical Activity" OR "Activities, Physical" OR "Activity, Physical" OR "Physical Activities") OR AB ("Exercise" OR "Exercises" OR "Exercise, Physical" OR "Exercises, Physical" OR "Physical Exercise" OR "Physical Exercises" OR "Exercise, Aerobic" OR "Aerobic Exercise" OR "Aerobic Exercises" OR "Exercises, Aerobic" OR "Exercise, Isometric" OR "Exercises, Isometric" OR "Isometric Exercises" OR "Isometric Exercise" OR "Acute Exercise" OR "Acute Exercises" OR "Exercise, Acute" OR "Exercises, Acute" OR "Exercise Training" OR "Exercise Trainings" OR "Training, Exercise" OR "Trainings, Exercise" OR "Physical Activity" OR "Activities, Physical" OR "Activity, Physical" OR "Physical Activities") |
| **#3** | SU (Depression) OR TI ("Depression" OR "Depressive Symptoms" OR "Depressive Symptom" OR "Symptom, Depressive" OR "Emotional Depression" OR "Depression, Emotional") OR AB ("Depression" OR "Depressive Symptoms" OR "Depressive Symptom" OR "Symptom, Depressive" OR "Emotional Depression" OR "Depression, Emotional") |
| **#4** | SU (Anxiety) OR TI ("Anxiety" OR "Angst" OR "Nervousness" OR "Hypervigilance" OR "Social Anxiety" OR "Anxieties, Social" OR "Anxiety, Social" OR "Social Anxieties" OR "Anxiousness") OR AB ("Anxiety" OR "Angst" OR "Nervousness" OR "Hypervigilance" OR "Social Anxiety" OR "Anxieties, Social" OR "Anxiety, Social" OR "Social Anxieties" OR "Anxiousness") |
| **#5** | SU (Quality of Life) OR TI ("Quality of Life" OR "Life Quality" OR "Health-Related Quality Of Life" OR "Health Related Quality Of Life" OR "HRQOL") OR AB ("Quality of Life" OR "Life Quality" OR "Health-Related Quality Of Life" OR "Health Related Quality Of Life" OR "HRQOL") |
| **#6** | #3 OR #4 OR #5 |
| **#7** | #1 AND #2 AND #6 |
| ****ProQuest 274 Results**** | |
| **#1** | mainsubject(Renal Dialysis) OR title("Renal Dialysis" OR "Dialyses, Renal" OR "Renal Dialyses" OR "Dialysis, Renal" OR "Hemodialysis" OR "Hemodialyses" OR "Dialysis, Extracorporeal" OR "Dialyses, Extracorporeal" OR "Extracorporeal Dialyses" OR "Extracorporeal Dialysis" ) OR abstract("Renal Dialysis" OR "Dialyses, Renal" OR "Renal Dialyses" OR "Dialysis, Renal" OR "Hemodialysis" OR "Hemodialyses" OR "Dialysis, Extracorporeal" OR "Dialyses, Extracorporeal" OR "Extracorporeal Dialyses" OR "Extracorporeal Dialysis" ) |
| **#2** | [mainsubject(Exercise) OR title("Exercise" OR "Exercises" OR "Exercise, Physical" OR "Exercises, Physical" OR "Physical Exercise" OR "Physical Exercises" OR "Exercise, Aerobic" OR "Aerobic Exercise" OR "Aerobic Exercises" OR "Exercises, Aerobic" OR "Exercise, Isometric" OR "Exercises, Isometric" OR "Isometric Exercises" OR "Isometric Exercise" OR "Acute Exercise" OR "Acute Exercises" OR "Exercise, Acute" OR "Exercises, Acute" OR "Exercise Training" OR "Exercise Trainings" OR "Training, Exercise" OR "Trainings, Exercise" OR "Physical Activity" OR "Activities, Physical" OR "Activity, Physical" OR "Physical Activities" ) OR abstract("Exercise" OR "Exercises" OR "Exercise, Physical" OR "Exercises, Physical" OR "Physical Exercise" OR "Physical Exercises" OR "Exercise, Aerobic" OR "Aerobic Exercise" OR "Aerobic Exercises" OR "Exercises, Aerobic" OR "Exercise, Isometric" OR "Exercises, Isometric" OR "Isometric Exercises" OR "Isometric Exercise" OR "Acute Exercise" OR "Acute Exercises" OR "Exercise, Acute" OR "Exercises, Acute" OR "Exercise Training" OR "Exercise Trainings" OR "Training, Exercise" OR "Trainings, Exercise" OR "Physical Activity" OR "Activities, Physical" OR "Activity, Physical" OR "Physical Activities" )](https://www.proquest.com/recentsearches.recentsearchtabview.recentsearchesgridview.scrolledrecentsearchlist.checkdbssearchlink:rerunsearch/CF84A42365034BE6PQ/None/$N?_csrf=a130825e-b234-4886-9799-307bb3f9859e&site=healthcomplete&t:ac=RecentSearches) |
| **#3** | mainsubject(Depression) OR title("Depression" OR "Depressive Symptoms" OR "Depressive Symptom" OR "Symptom, Depressive" OR "Emotional Depression" OR "Depression, Emotional" ) OR abstract("Depression" OR "Depressive Symptoms" OR "Depressive Symptom" OR "Symptom, Depressive" OR "Emotional Depression" OR "Depression, Emotional" ) |
| **#4** | mainsubject(Anxiety) OR title("Anxiety" OR "Angst" OR "Nervousness" OR "Hypervigilance" OR "Social Anxiety" OR "Anxieties, Social" OR "Anxiety, Social" OR "Social Anxieties" OR "Anxiousness") OR abstract("Anxiety" OR "Angst" OR "Nervousness" OR "Hypervigilance" OR "Social Anxiety" OR "Anxieties, Social" OR "Anxiety, Social" OR "Social Anxieties" OR "Anxiousness") |
| **#5** | [mainsubject(Quality of Life) OR title("Quality of Life" OR "Life Quality" OR "Health-Related Quality Of Life" OR "Health Related Quality Of Life" OR "HRQOL" ) OR abstract("Quality of Life" OR "Life Quality" OR "Health-Related Quality Of Life" OR "Health Related Quality Of Life" OR "HRQOL" )](https://www.proquest.com/recentsearches.recentsearchtabview.recentsearchesgridview.scrolledrecentsearchlist.checkdbssearchlink:rerunsearch/FCE8E64C886C417BPQ/None/$N?_csrf=a130825e-b234-4886-9799-307bb3f9859e&site=healthcomplete&t:ac=RecentSearches) |
| **#6** | #3 OR #4 OR #5 |
| **#7** | #1 AND #2 AND #6 |
| ****Wangfang Database 887 Results**** | |
| **#1** | 主题: 运动 or 训练 or 锻炼 |
| **#2** | 主题: 透析 or 血透 or 血液透析 or 血液净化 |
| **#3** | 主题: 抑郁 or 焦虑 or 生活质量 |
| **#4** | 摘要: 随机 or 随机对照 or 随机分组 |
| **#5** | #1 AND #2 AND #3 AND #4 |
| ****China Science and Technology Journal Database(VIP) 119 Results**** | |
| **#1** | 题名或关键词: 运动 or 训练 or 锻炼 |
| **#2** | 题名或关键词: 透析 or 血透 or 血液透析 or 血液净化 |
| **#3** | 题名或关键词: 抑郁 or 焦虑 or 生活质量 |
| **#4** | 摘要: 随机 or 随机对照 or 随机分组 |
| **#5** | #1 AND #2 AND #3 AND #4 |
| ****China National Knowledge Infrastructure(CNKI) 259 Results**** | |
| **#1** | 主题: 运动 + 训练 + 锻炼 |
| **#2** | 主题: 透析 + 血透 + 血液透析 + 血液净化 |
| **#3** | 主题: 抑郁 + 焦虑 + 生活质量 |
| **#4** | 摘要: 随机 + 随机对照 + 随机分组 |
| **#5** | #1 AND #2 AND #3 AND #4 |
| ****China Biology Medicine disc(CBM) 430 Results**** | |
| **#1** | "运动"[常用字段:智能] OR "训练"[常用字段:智能] OR "锻炼"[常用字段:智能] |
| **#2** | "透析"[常用字段:智能] OR "血透"[常用字段:智能] OR "血液透析"[常用字段:智能] OR "血液净化"[常用字段:智能] |
| **#3** | "抑郁"[常用字段:智能] OR "焦虑"[常用字段:智能] OR "生活质量"[常用字段:智能] |
| **#4** | "随机"[常用字段:智能] OR "随机对照"[常用字段:智能] OR "随机分组"[常用字段:智能] |
| **#5** | #1 AND #2 AND #3 AND #4 |

**Supplementary Table 2.** Excluded studies

|  | Title | Reasons for exclusion |
| --- | --- | --- |
| 1 | Afshar R, Emany A, Saremi A, Shavandi N, Sanavi S. Effects of intradialytic aerobic training on sleep quality in hemodialysis patients. Iran J Kidney Dis. 2011;5(2):119-123. | Irrelevant outcome |
| 2 | Aoike DT, Baria F, Kamimura MA, Ammirati A, Cuppari L. Home-based versus center-based aerobic exercise on cardiopulmonary performance, physical function, quality of life and quality of sleep of overweight patients with chronic kidney disease. Clin Exp Nephrol. 2018;22(1):87-98. | Ineligible population |
| 3 | Bacalso LV, Cuenza L, Ebba E, King A. Effects of intradialytic exercise training on functional capacity and quality of life among hemodialysis patients at a tertiary cardiac center in the Philippines. Journal of the Hong Kong College of Cardiology. 2020;28(2):84. | Ineligible intervention |
| 4 | Zhang F, Liao J, Zhang W, et al. Effects of Baduanjin Exercise on Physical Function and Health-Related Quality of Life in Peritoneal Dialysis Patients: A Randomized Trial. Front Med (Lausanne). 2021;8:789521. | Ineligible population |
| 5 | Bennett PN, Parsons T, Ben-Moshe R, et al. Intradialytic Laughter Yoga therapy for haemodialysis patients: a pre-post intervention feasibility study. BMC Complement Altern Med. 2015;15:176. | Ineligible study design |
| 6 | Bennett PN, Daly RM, Fraser SF, et al. The impact of an exercise physiologist coordinated resistance exercise program on the physical function of people receiving hemodialysis: a stepped wedge randomised control study. BMC Nephrol. 2013;14:204. | Irrelevant outcome |
| 7 | Baggetta R, D'Arrigo G, Torino C, et al. Effect of a home based, low intensity, physical exercise program in older adults dialysis patients: a secondary analysis of the EXCITE trial. BMC Geriatr. 2018;18(1):248. | Irrelevant outcome |
| 8 | Goldberg AP, Geltman EM, Hagberg JM, et al. Therapeutic benefits of exercise training for hemodialysis patients. Kidney Int Suppl. 1983;16:S303-S309. | Irrelevant outcome |
| 9 | Carney RM, Templeton B, Hong BA, et al. Exercise training reduces depression and increases the performance of pleasant activities in hemodialysis patients. Nephron. 1987;47(3):194-198. | Insufficient data |
| 10 | Tsai TJ, Lai JS, Lee SH, et al. Breathing-coordinated exercise improves the quality of life in hemodialysis patients. J Am Soc Nephrol. 1995;6(5):1392-1400. | Insufficient data |
| 11 | Martins do Valle F, Valle Pinheiro B, Almeida Barros AA, et al. Effects of intradialytic resistance training on physical activity in daily life, muscle strength, physical capacity and quality of life in hemodialysis patients: a randomized clinical trial. Disabil Rehabil. 2020;42(25):3638-3644. | Insufficient data |
| 12 | Painter P, Carlson L, Carey S, Paul SM, Myll J. Physical functioning and health-related quality-of-life changes with exercise training in hemodialysis patients. Am J Kidney Dis. 2000;35(3):482-492. | Insufficient data |
| 13 | Painter P, Carlson L, Carey S, Paul SM, Myll J. Low-functioning hemodialysis patients improve with exercise training. Am J Kidney Dis. 2000;36(3):600-608. | Ineligible study design |
| 14 | Tawney KW, Tawney PJ, Hladik G, et al. The life readiness program: a physical rehabilitation program for patients on hemodialysis. Am J Kidney Dis. 2000;36(3):581-591. | Ineligible intervention |
| 15 | Suh MR, Jung HH, Kim SB, Park JS, Yang WS. Effects of regular exercise on anxiety, depression, and quality of life in maintenance hemodialysis patients. Ren Fail. 2002;24(3):337-345. | Ineligible study design |
| 16 | Cheng YY, Wong YF, Chu BYC, Lam WO, Ho YW. Rehabilitating a dialysis patient. Peritoneal Dialysis International. 2003;23(Suppl 2). | Ineligible study design |
| 17 | Kosmadakis GC, John SG, Clapp EL, et al. Benefits of regular walking exercise in advanced pre-dialysis chronic kidney disease. Nephrol Dial Transplant. 2012;27(3):997-1004. | Ineligible population |
| 18 | Kusek JW, Greene P, Wang SR, et al. Cross-sectional study of health-related quality of life in African Americans with chronic renal insufficiency: the African American Study of Kidney Disease and Hypertension Trial. Am J Kidney Dis. 2002;39(3):513-524. | Ineligible study design |
| 19 | Johansen KL, Painter PL, Sakkas GK, Gordon P, Doyle J, Shubert T. Effects of resistance exercise training and nandrolone decanoate on body composition and muscle function among patients who receive hemodialysis: A randomized, controlled trial. J Am Soc Nephrol. 2006;17(8):2307-2314. | Ineligible intervention |
| 20 | Koh KP, Fassett RG, Sharman JE, Coombes JS, Williams AD. Intradialytic versus home-based exercise training in hemodialysis patients: a randomised controlled trial. BMC Nephrol. 2009;10:2. Published 2009 Jan 29. | Irrelevant outcome |
| 21 | Chang Y, Cheng SY, Lin M, Gau FY, Chao YF. The effectiveness of intradialytic leg ergometry exercise for improving sedentary life style and fatigue among patients with chronic kidney disease: a randomized clinical trial. Int J Nurs Stud. 2010;47(11):1383-1388. | Irrelevant outcome |
| 22 | Cheema BS, Abas H, Smith BC, et al. Effect of resistance training during hemodialysis on circulating cytokines: a randomized controlled trial. Eur J Appl Physiol. 2011;111(7):1437-1445. | Irrelevant outcome |
| 23 | Barcellos FC, Santos IS, Mielke GI, del Vecchio FB, Hallal PC. Effects of exercise on kidney function among non-diabetic patients with hypertension and renal disease: randomized controlled trial. BMC Nephrol. 2012;13:90. | Ineligible population |
| 24 | Rambod M, Pourali-Mohammadi N, Pasyar N, Rafii F, Sharif F. The effect of Benson's relaxation technique on the quality of sleep of Iranian hemodialysis patients: a randomized trial. Complement Ther Med. 2013;21(6):577-584. | Ineligible intervention |
| 25 | Choudhary SK, Zagade TB. Efficacy of Deep Breathing Exercise and Range of Motion Exercise on Depression, Anxiety, and Stress in Patients with Chronic Kidney Disease Undergoing Hemodialysis. J Pharm Bioallied Sci. 2025;17(Suppl 3):S2566-S2568. | Insufficient data |
| 26 | Santos MAD, Conceição APD, Ferretti-Rebustini REL, Ciol MA, Heithkemper MM, Cruz DALMD. Non-pharmacological interventions for sleep and quality of life: a randomized pilot study. Rev Lat Am Enfermagem. 2018;26:e3079. | Ineligible study design |
| 27 | Nonoyama ML, Brooks D, Ponikvar A, et al. Exercise program to enhance physical performance and quality of life of older hemodialysis patients: a feasibility study. Int Urol Nephrol. 2010;42(4):1125-1130. | Ineligible study design |
| 28 | Paluchamy T, Vaidyanathan R. Effectiveness of intradialytic exercise on dialysis adequacy, physiological parameters, biochemical markers and quality of life - A pilot study. Saudi J Kidney Dis Transpl. 2018;29(4):902-910. | Ineligible study design |
| 29 | Moeinzadeh F, Shahidi S, Shahzeidi S. Evaluating the effect of intradialytic cycling exercise on quality of life and recovery time in hemodialysis patients: A randomized clinical trial. J Res Med Sci. 2022;27:84. | Insufficient data |
| 30 | Frih B, Jaafar H, Mkacher W, Ben Salah Z, Hammami M, Frih A. The Effect of Interdialytic Combined Resistance and Aerobic Exercise Training on Health Related Outcomes in Chronic Hemodialysis Patients: The Tunisian Randomized Controlled Study. Front Physiol. 2017;8:288. | Irrelevant outcome |
| 31 | Motedayen Z, Nehrir B, Tayebi A, Ebadi A, Einollahi B. The effect of the physical and mental exercises during hemodialysis on fatigue: a controlled clinical trial. Nephrourol Mon. 2014;6(4):e14686. | Irrelevant outcome |
| 32 | Güvener YÖ, Koç Z. The effect of breathing exercises on pain, sleep, and symptom management in patients undergoing hemodialysis: a randomized controlled trial. Sleep Breath. 2025;29(2):170. | Irrelevant outcome |
| 33 | Maniam R, Subramanian P, Singh SK, Lim SK, Chinna K, Rosli R. Preliminary study of an exercise programme for reducing fatigue and improving sleep among long-term haemodialysis patients. Singapore Med J. 2014;55(9):476-482. | Insufficient data |
| 34 | Figueiredo PHS, Lima MMO, Costa HS, et al. Effects of the inspiratory muscle training and aerobic training on respiratory and functional parameters, inflammatory biomarkers, redox status and quality of life in hemodialysis patients: A randomized clinical trial. PLoS One. 2018;13(7):e0200727. | Insufficient data |
| 35 | Uchiyama K, Washida N, Morimoto K, et al. Home-based Aerobic Exercise and Resistance Training in Peritoneal Dialysis Patients: A Randomized Controlled Trial. Sci Rep. 2019;9(1):2632. | Ineligible population |
| 36 | Dziubek W, Kowalska J, Kusztal M, et al. The Level of Anxiety and Depression in Dialysis Patients Undertaking Regular Physical Exercise Training--a Preliminary Study. Kidney Blood Press Res. 2016;41(1):86-98. | Ineligible study design |
| 37 | Myers J, Chan K, Chen Y, et al. Effect of a Home-Based Exercise Program on Indices of Physical Function and Quality of Life in Elderly Maintenance Hemodialysis Patients. Kidney Blood Press Res. 2021;46(2):196-206. | Insufficient data |
| 38 | McGregor G, Ennis S, Powell R, et al. Feasibility and effects of intra-dialytic low-frequency electrical muscle stimulation and cycle training: A pilot randomized controlled trial. PLoS One. 2018;13(7):e0200354. | Ineligible study design |
| 39 | Romeu-Perales M, Segura-Ortí E, Cana-Poyatos A, et al. The Effect of Intradialytic Exercise Using Virtual Reality on the Body Composition of Patients with Chronic Kidney Disease. Nutrients. 2024;16(12):1968. | Irrelevant outcome |
| 40 | Magnard J, Deschamps T, Cornu C, Paris A, Hristea D. Effects of a six-month intradialytic physical ACTIvity program and adequate NUTritional support on protein-energy wasting, physical functioning and quality of life in chronic hemodialysis patients: ACTINUT study protocol for a randomised controlled trial. BMC Nephrol. 2013;14:259. | Ineligible study design |
| 41 | Zhao C, Ma H, Yang L, Xiao Y. Long-term bicycle riding ameliorates the depression of the patients undergoing hemodialysis by affecting the levels of interleukin-6 and interleukin-18. Neuropsychiatr Dis Treat. 2016;13:91-100. | Ineligible intervention |
| 42 | Rahimimoghadam Z, Rahemi Z, Sadat Z, Mirbagher Ajorpaz N. Pilates exercises and quality of life of patients with chronic kidney disease. Complement Ther Clin Pract. 2019;34:35-40. | Ineligible population |
| 43 | Barbosa, P.L., Blumer, E.A., Oliveira, J.C.S. et al. A multicomponent exercise program improves functional capacity and respiratory muscle strength in hemodialysis patients: a randomized clinical trial. Sport Sci Health 19, 1217–1225 (2023). | Irrelevant outcome |
| 44 | Fathi M, Hejazi K. The effect of six months aerobic exercise during dialysis on liver enzymes, cystatin C and quality of life of hemodialysis patients. J Sports Med Phys Fitness. 2021;61(11):1515-1522. | Insufficient data |
| 45 | Mallamaci F, Bratsiakou A, D'Arrigo G, et al. Sex Differences in the EXerCise Introduction to Enhance Performance in Dialysis Trial. Clin J Am Soc Nephrol. 2025;20(8):1072-1078. | Irrelevant outcome |
| 46 | Sajjadi SL, Ghafourifard M, Khosroshahi HT. The effect of individualized education on learning needs of patients undergoing hemodialysis: a randomized controlled clinical trial. BMC Nephrol. 2024;25(1):452. | Irrelevant outcome |
| 47 | Gravina EPL, Pinheiro BV, da Silva Jesus LA, et al. Effects of long-term aerobic training and detraining on functional capacity and quality of life in hemodialysis patients: A pilot study. Int J Artif Organs. 2020;43(6):411-415. | Ineligible study design |
| 48 | Tao X, Chow SK, Wong FK. A nurse-led case management program on home exercise training for hemodialysis patients: A randomized controlled trial. Int J Nurs Stud. 2015;52(6):1029-1041. | Insufficient data |
| 49 | Sovatzidis A, Chatzinikolaou A, Fatouros IG, et al. Intradialytic Cardiovascular Exercise Training Alters Redox Status, Reduces Inflammation and Improves Physical Performance in Patients with Chronic Kidney Disease. Antioxidants (Basel). 2020;9(9):868. | Insufficient data |
| 50 | Kirkman DL, Mullins P, Junglee NA, Kumwenda M, Jibani MM, Macdonald JH. Anabolic exercise in haemodialysis patients: a randomised controlled pilot study. J Cachexia Sarcopenia Muscle. 2014;5(3):199-207. | Ineligible study design |
| 51 | Guo D, Fadel WF, Cranor AA, Moe SM, Avin KG, Moorthi RN. Physical activity in patients undergoing dialysis: a pilot study: PO1755. Journal of the American Society of Nephrology. 2021;32(10S):543-544. | Ineligible study design |
| 52 | Usui N, Uehata A, Nakata J, et al. Effect of blood volume change related to intensity of intradialytic aerobic exercise on hemodialysis adequacy: a pilot study. Int Urol Nephrol. 2022;54(6):1427-1434. | Ineligible study design |
| 53 | Matsumoto Y, Furuta A, Furuta S, et al. The impact of pre-dialytic endurance training on nutritional status and quality of life in stable hemodialysis patients (Sawada study). Ren Fail. 2007;29(5):587-593. | Insufficient data |
| 54 | Li WY, Yeh JC, Cheng CC, et al. Digital health interventions to promote healthy lifestyle in hemodialysis patients: an interventional pilot study. Sci Rep. 2024;14(1):2849. | Ineligible study design |
| 55 | Takamatsu K, Shike T, Kaneda Y, et al. Physical and psychological effects of a long-term supervised self-exercise program during hemodialysis in elderly dialysis patients: A single-site pilot study in a Japanese community setting. Medicine (Baltimore). 2024;103(29):e38963. | Ineligible study design |
| 56 | Moug SJ, Grant S, Creed G, Boulton Jones M. Exercise during haemodialysis: West of Scotland pilot study. Scott Med J. 2004;49(1):14-17. | Ineligible study design |
| 57 | Anding K, Bär T, Trojniak-Hennig J, et al. A structured exercise programme during haemodialysis for patients with chronic kidney disease: clinical benefit and long-term adherence. BMJ Open. 2015;5(8):e008709. | Ineligible study design |
| 58 | Suhardjono, Umami V, Tedjasukmana D, Setiati S. The effect of intradialytic exercise twice a week on the physical capacity, inflammation, and nutritional status of dialysis patients: A randomized controlled trial. Hemodial Int. 2019;23(4):486-493. | Insufficient data |
| 59 | Giannaki CD, Sakkas GK, Karatzaferi C, et al. Effect of exercise training and dopamine agonists in patients with uremic restless legs syndrome: a six-month randomized, partially double-blind, placebo-controlled comparative study. BMC Nephrol. 2013;14:194. | Ineligible intervention |
| 60 | Manfredini F, Mallamaci F, D'Arrigo G, et al. Exercise in Patients on Dialysis: A Multicenter, Randomized Clinical Trial. J Am Soc Nephrol. 2017;28(4):1259-1268. | Insufficient data |
| 61 | Esteve Simo V, Junqué Jiménez A, Moreno Guzmán F, et al. Benefits of a low intensity exercise programme during haemodialysis sessions in elderly patients. Nefrologia. 2015;35(4):385-394. | Ineligible study design |
| 62 | Ribeiro HS, Cunha VA, Dourado GÍ, et al. Implementing a resistance training programme for patients on short daily haemodialysis: A feasibility study. J Ren Care. 2023;49(2):125-133. | Ineligible study design |
| 63 | Hornik B, Duława J, Durmała J. Metabolic Syndrome and Psychological Effects of Exercise in Hemodialysis Patients. Int J Environ Res Public Health. 2021;18(22):11952. | Ineligible study design |
| 64 | Lorenz EC, Hickson LJ, Hogan MC, Kennedy CC. Examining the safety and effectiveness of a 4-week supervised exercise intervention in the treatment of frailty in patients with chronic kidney disease. Clin Kidney J. 2023;16(11):2003-2010. | Ineligible study design |
| 65 | Malagoni AM, Catizone L, Mandini S, et al. Acute and long-term effects of an exercise program for dialysis patients prescribed in hospital and performed at home. J Nephrol. 2008;21(6):871-878. | Insufficient data |
| 66 | Hsieh RL, Huang HY, Chen SC, et al. Changes in physical functional performance and quality of life in hemodialysis patients in Taiwan: a preliminary study. J Nephrol. 2010;23(1):41-48. | Ineligible study design |
| 67 | Doyle A, Chalmers K, Chinn DJ, McNeill F, Dall N, Grant CH. The utility of whole body vibration exercise in haemodialysis patients: a pilot study. Clin Kidney J. 2017;10(6):822-829. | Ineligible study design |
| 68 | Tentori F, Elder SJ, Thumma J, et al. Physical exercise among participants in the Dialysis Outcomes and Practice Patterns Study (DOPPS): correlates and associated outcomes. Nephrol Dial Transplant. 2010;25(9):3050-3062. | Ineligible study design |
| 69 | Darawad MW, Khalil AA. Jordanian dialysis patients' perceived exercise benefits and barriers: a correlation study. Rehabil Nurs. 2013;38(6):315-322. | Ineligible study design |
| 70 | Watson EL, Greening NJ, Viana JL, et al. Progressive Resistance Exercise Training in CKD: A Feasibility Study. Am J Kidney Dis. 2015;66(2):249-257. | Ineligible study design |
| 71 | Alshammari B, Edison JS, Alkubati SA, et al. Effectiveness of exercise in reducing symptom burden among hemodialysis patients: a non-pharmacological intervention approach. Front Public Health. 2025;13:1580689. | Ineligible study design |
| 72 | Almutary H, AlShammari N. Treatment of depression and poor quality of life through breathing training in hemodialysis patients. BMC Nephrol. 2025;26(1):16. | Ineligible study design |
| 73 | Young HML, March DS, Highton PJ, et al. Exercise for people living with frailty and receiving haemodialysis: a mixed-methods randomised controlled feasibility study. BMJ Open. 2020;10(11):e041227. | Ineligible study design |
| 74 | Yogalakshmi S, Sasikala D, Varughese S, Sundararajan V. Integrated Dialysis Nursing Intervention for Ameliorating Fatigue in Hemodialysis Patients. Ethiop J Health Sci. 2024;34(5):389-396. | Ineligible study design |
| 75 | Vyas R, Malik AI, Sharma S, Rana P, Bahuguna K, Renuka. Effectiveness of leg exercises on muscle cramps among patients on haemodialysis in selected Government Hospitals, Dehradun, Uttrakhand. Journal of Cardiovascular Disease Research. 2024;15(6):849-856. | Ineligible study design |
| 76 | Wu Y, He Q, Yin X, He Q, Cao S, Ying G. Effect of individualized exercise during maintenance haemodialysis on exercise capacity and health-related quality of life in patients with uraemia. J Int Med Res. 2014;42(3):718-727. | Insufficient data |
| 77 | Masoudi R, Lotfizade M, Gheysarieha MR, Rabiei L. Evaluating the effect of Pender's health promotion model on self-efficacy and treatment adherence behaviors among patients undergoing hemodialysis. J Educ Health Promot. 2020;9:197. | Ineligible study design |
| 78 | Alishahi M, Mazloum SR, Mohajer S, Namazinia M. The effect of recreational therapy application on fatigue in hemodialysis patients: a randomized clinical trial. BMC Nephrol. 2024;25(1):368. | Irrelevant outcome |
| 79 | Sari RY, Kartini Y, Faizah I, Rohmawati R, Hasina SN, Putri RA. Combination of AROM with deep breathing exercise against fatigue and quality of life of hemodialysis patients; an experimental study. Journal of Nephropharmacology. 2024;13(1). | Ineligible study design |
| 80 | Swapna Mary A, Chiranjeevi V. Efficacy of intradialytic stretching exercise on health-related quality of life among patients undergoing haemodialysis. Journal of Clinical and Diagnostic Research. 2019;13(4):LC01-LC04. | Ineligible study design |
| 81 | de Lima MC, Cicotoste Cde L, Cardoso Kda S, Forgiarini LA Jr, Monteiro MB, Dias AS. Effect of exercise performed during hemodialysis: strength versus aerobic. Ren Fail. 2013;35(5):697-704. | Insufficient data |
| 82 | Salhab N, Alrukhaimi M, Kooman J, et al. Effect of Intradialytic Exercise on Hyperphosphatemia and Malnutrition. Nutrients. 2019;11(10):2464. | Ineligible study design |
| 83 | Dziubek W, Bulińska K, Kusztal M, et al. Evaluation of Exercise Tolerance in Dialysis Patients Performing Tai Chi Training: Preliminary Study. Evid Based Complement Alternat Med. 2016;2016:5672580. | Ineligible study design |
| 84 | Oliveros R MS, Avendaño M, Bunout D, et al. Estudio piloto sobre entrenamiento físico durante hemodiálisis [A pilot study on physical training of patients in hemodialysis]. Rev Med Chil. 2011;139(8):1046-1053. | Ineligible study design |
| 85 | Devagourou A, Sharma KK, Yadav RK, Gupta VP, Kalaivani M. An experimental study to evaluate the effect of low-intensity intradialytic exercises on serum urea, creatinine, and fatigue of chronic kidney disease patients undergoing hemodialysis. Saudi J Kidney Dis Transpl. 2021;32(5):1253-1259. | Ineligible study design |

**Supplementary Table 3.** Descriptions of intervention

| **Study** | **Country** | **Timing** | **Load** | **Volume** | **Equipment** | **Characteristics of intervention** |
| --- | --- | --- | --- | --- | --- | --- |
| Chen  et al.2010 | US | Intradialytic | Progressive | <90 | Without | Low-intensity lower extremity strength training was performed utilizing ankle weights. The protocol comprised four distinct exercises, with each exercise executed for 2 sets of 8 repetitions. |
| Deus  et al.2021 | Brazil | Non-intradialytic | Progressive | >120 | Without | Strength training was performed utilizing dumbbells, elastic bands, and bodyweight. The protocol comprised 12 distinct exercises, each executed for 3 sets of 8–12 repetitions. |
| Dobsak et al.2012 | Czech | Intradialytic | Progressive | 90-120 | With | Aerobic training was performed utilizing a bedside stationary bicycle equipped with a power meter. During the initial five weeks, sessions lasted 20 minutes. Subsequently, the training volume was progressed to 2 sets of 20 minutes. |
| Feng et al.2025 | China | Intradialytic | Progressive | 90-120 | Without | Strength training was performed utilizing elastic bands. The protocol consisted of four distinct exercises, each executed for 3 sets of 20–30 repetitions. |
| Fu et al.2021 | China | Non-intradialytic | Non-progressive | 90-120 | Without | Patients engaged in Qigong Baduanjin training. The complete routine comprised the Preparatory Form, the Closing Form, and eight distinct movements, performed for 2–3 cycles per session. |
| Giannaki et al.2013 | Greece | Intradialytic | Progressive | >120 | With | Aerobic cycling training was performed utilizing a recumbent cycle ergometer, with the workload adjusted every four weeks. |
| Huang et al.2020 | China | Intradialytic | Progressive | 90-120 | With | Training was conducted utilizing a dual-mode cycle ergometer featuring both aerobic and strength settings. Sessions commenced with aerobic exercise followed by strength training, with the time allocation for each modality adjusted progressively throughout the intervention period. |
| Jamshidpour et al.2020 | Iran | Intradialytic | Progressive | <90 | With | Hip muscle strength training was performed in bed using free weight equipment, and aerobic training was performed using a bedside stationary bicycle paired with a power meter. The training time for aerobic training increased as the treatment course progressed. |
| Kim et al.2023 | Korea | Intradialytic | Progressive | 90-120 | With | Aerobic training was performed using in-bed pedal exercise equipment paired with a dynamometer. Before formal training, a sports education course was also conducted once. |
| Kouidi et al.1997 | Greece | Non-intradialytic | Progressive | >120 | With | Aerobic cycling exercise was performed using a stationary bicycle paired with a portable telemetry spirometer. Later, swimming and jogging on non-dialysis days were also added. |
| Kouidi et al.2010 | Greece | Intradialytic | Progressive | >120 | With | Aerobic and strength training were performed using in-bed pedal exercise equipment with motor power support, as well as elastic bands or free weights. Strength training was added in the later stage of training. |
| Li et al.2025 | China | Intradialytic | Progressive | 90-120 | Without | Patients first performed 15 minutes of simple in-bed aerobic calisthenics, including 4 movements. On the non-dialysis side, a grip ball was used, holding for 2 seconds and relaxing for 2 seconds, 10 repetitions per set, for 10 sets. Elastic bands were fixed on both calves to perform knee extension, hip abduction, and hip flexion training. Each movement was done 10 times per leg, for 3 sets. |
| Li et al.2024 | China | Intradialytic | Non-progressive | 90-120 | With | Aerobic cycling training was conducted utilizing a cycle ergometer featuring both active and passive modes, coupled with a portable spirometer. |
| Li et al.2024 | China | Intradialytic | Non-progressive | 90-120 | Without | Sandbags were positioned on the patient's upper abdomen to impose an inspiratory load. The weight was titrated based on individual tolerance, capped at 2 kg, to prevent diaphragmatic fatigue. |
| Liu et al.2023 | China | Intradialytic | Progressive | 90-120 | With | Aerobic exercise intervention was conducted utilizing a professional-grade treadmill integrated with a cardiopulmonary exercise testing system. The protocol was structured into three distinct phases: warm-up, formal training, and cool-down, with exercise intensity titrated according to the progression of the treatment stages. |
| Marieke et al.2005 | Netherlands | Intradialytic+Non-intradialytic | Non-progressive | 90-120 | With | The intervention comprised pre-dialysis strength training conducted at the center's gymnasium, followed by intradialytic cycling utilizing a custom-fabricated device developed by a professional technical team. |
| Maynard et al.2019 | Brazil | Intradialytic | Progressive | 90-120 | With | Endurance and strength training were conducted utilizing Virtual Reality equipment, augmented by weighted sandbags, elastic bands, and balls. The VR game scenarios and training difficulty were adjusted biweekly. Furthermore, the regimen was supplemented with aerobic training via a stationary bicycle. |
| Ouzouni et al.2009 | Greece | Intradialytic | Progressive | >120 | With | Aerobic and strength training were performed utilizing a bedside cycle ergometer, elastic bands, and weighted equipment. Blood pressure, oxygen uptake, and electrocardiography were continuously monitored during sessions. Exercise intensity was incrementally increased as the intervention progressed. |
| Pereira et al.2022 | Brazil | Intradialytic | Progressive | 90-120 | With | Aerobic training was conducted utilizing a cycle ergometer, with cycling speed progressively increased throughout the intervention. Vital signs were monitored at 10-minute intervals during each session. |
| Prestes et al.2025 | Brazil | Non-intradialytic | Progressive | >120 | Without | Traditional strength training was implemented, comprising 12 distinct exercises with a specific emphasis on unilateral upper extremity movements to safeguard the arteriovenous fistula. The protocol prescribed 3 sets of 8–12 repetitions, with a 2-minute rest interval between sets. |
| Prestes et al.2025 | Brazil | Non-intradialytic | Progressive | >120 | Without | Cluster set strength training was implemented, similarly comprising 12 exercises with an emphasis on unilateral upper extremity movements to safeguard the arteriovenous fistula. While the protocol maintained 3 sets, it incorporated a 15-second intra-set rest interval after every 4 repetitions. |
| Rezaei  et al.2015 | Iran | Non-intradialytic | Non-progressive | 90-120 | Without | Instruction was delivered via face-to-face sessions and educational posters until patients demonstrated proficiency in the exercise techniques. The home-based regimen primarily targeted the lower back and abdominal musculature, requiring no specialized equipment. |
| Rosa et al.2018 | Brazil | Intradialytic | Progressive | 90-120 | Without | Strength training was conducted utilizing elastic bands and free weights. The protocol comprised 11 specific exercises targeting both the upper and lower extremities, with each exercise executed for 2 sets of 15–20 repetitions. Exercise intensity was progressively increased based on patient improvement. |
| Samara  et al.2016 | Greece | Non-intradialytic | Progressive | >120 | With | Aquatic exercise was implemented, predominantly consisting of swimming. Vital signs were monitored continuously throughout the sessions utilizing specialized monitoring equipment. |
| Siou-Hung et al.2015 | China | Non-intradialytic | Non-progressive | <90 | Without | Respiratory training was conducted, guided by pre-recorded instructional audio on respiratory techniques, vocal cues, and background music. The intervention focused primarily on deep, slow diaphragmatic respiration. |
| Song and Sohng.2012 | Korea | Non-intradialytic | Progressive | 90-120 | Without | Strength training was conducted utilizing elastic bands and weighted sandbags. The protocol comprised seven distinct exercises, each executed for 1–3 sets of 10–15 repetitions, with the workload incrementally increased as the intervention progressed. |
| Turon-Skrzypinska et al.2023 | Poland | Intradialytic | Non-progressive | <90 | With | Aerobic training was conducted utilizing immersive Virtual Reality technology, primarily comprising two game-based scenarios. Notably, the range of motion was restricted for the limb bearing the arteriovenous fistula. |
| Yu et al.2021 | China | Intradialytic | Non-progressive | >120 | With | Aerobic and strength training were conducted utilizing an in-bed cycle ergometer featuring active and passive modes, supplemented by sandbags and elastic bands. Specifically, the strength training targeted the non-access upper extremity and lower limb musculature. |
| Yuenyongchaiwat et al.2021 | Thailand | Intradialytic | Progressive | <90 | Without | Respiratory training was performed by increasing the load on the respiratory muscles. Each session consisted of three sets, with 15 inspiratory repetitions per set, and a rest of 60 seconds between sets. |

**Supplementary Table 4.** Meta-regression of the effectiveness of exercise training on depression

| **_ES** | **Coef.** | **Std.Err.** | **t** | **P>\|t\|** | **95%CI** | **I^2^** |
| --- | --- | --- | --- | --- | --- | --- |
| Region | -0.138 | 0.145 | -0.95 | 0.357 | 0.45;0.17 | 66.72% |
| Age | -0.700 | 0.180 | -3.88 | **0.002** | -1.09; -0.31 | 28.25% |
| Type | -0.003 | 0.088 | -0.03 | 0.975 | -0.19;0.19 | 68.30% |
| Duration | -0.663 | 0.204 | -3.24 | **0.006** | -1.10;-0.22 | 41.23% |
| Control | 0.052 | 0.152 | 0.34 | 0.739 | -0.27;0.38 | 67.09% |
| Timing | 0.264 | 0.179 | 1.48 | 0.162 | -0.12;0.65 | 65.48% |
| Specialized equipment | -0.181 | 0.236 | -0.77 | 0.455 | -0.69;0.32 | 68.31% |
| Scale | -0.044 | 0.086 | -0.51 | 0.616 | -0.23;0.14 | 64.54% |
| Load | 0.233 | 0.244 | 0.96 | 0.355 | -0.29;0.76 | 68.64% |
| Volume | -0.262 | 0.167 | -1.57 | 0.139 | -0.62;0.10 | 67.90% |

Abbreviations: **CI**, confidence interval

**Supplementary Table 5.** Meta-regression of the effectiveness of exercise training on anxiety

| **_ES** | **Coef.** | **Std.Err.** | **t** | **P>\|t\|** | **95%CI** | **I^2^** |
| --- | --- | --- | --- | --- | --- | --- |
| Region | 0.156 | 0.430 | 0.36 | 0.742 | -1.21, 1.52 | 74.97% |
| Duration | -0.761 | 0.208 | -3.66 | **0.035** | -1.42, -0.10 | 0.00% |
| Specialized equipment | 0.156 | 0.430 | 0.36 | 0.742 | -1.21, 1.52 | 74.97% |
| Load | -0.237 | 0.411 | -0.58 | 0.604 | -1.55, 1.07 | 74.73% |

Abbreviations: **CI**, confidence interval

**Supplementary Table 6.** Meta-regression of the effectiveness of exercise training on physical health-related quality of life

| **_ES** | **Coef.** | **Std.Err.** | **t** | **P>\|t\|** | **95%CI** | **I^2^** |
| --- | --- | --- | --- | --- | --- | --- |
| Region | -0.094 | 0.077 | -1.22 | 0.243 | -0.26, 0.07 | 36.20% |
| Age | 0.389 | 0.214 | 1.82 | 0.09 | -0.07, 0.85 | 27.62% |
| Type | -0.030 | 0.107 | -0.28 | 0.78 | -0.26, 0.20 | 43.25% |
| Duration | 0.359 | 0.310 | 1.16 | 0.266 | -0.31, 1.02 | 36.86% |
| Control | -0.005 | 0.124 | -0.04 | 0.97 | -0.27, 0.26 | 43.27% |
| Timing | -0.115 | 0.279 | -0.41 | 0.686 | -0.71, 0.48 | 42.44% |
| Specialized equipment | 0.415 | 0.198 | 2.09 | 0.055 | -0.01, 0.84 | 24.95% |
| Scale | -0.346 | 0.252 | -1.37 | 0.191 | -0.89, 0.19 | 35.30% |
| Load | -0.079 | 0.253 | -0.31 | 0.759 | -0.62, 0.46 | 43.15% |
| Volume | 0.279 | 0.148 | 1.88 | 0.081 | -0.04, 0.60 | 27.75% |

Abbreviations: **CI**, confidence interval


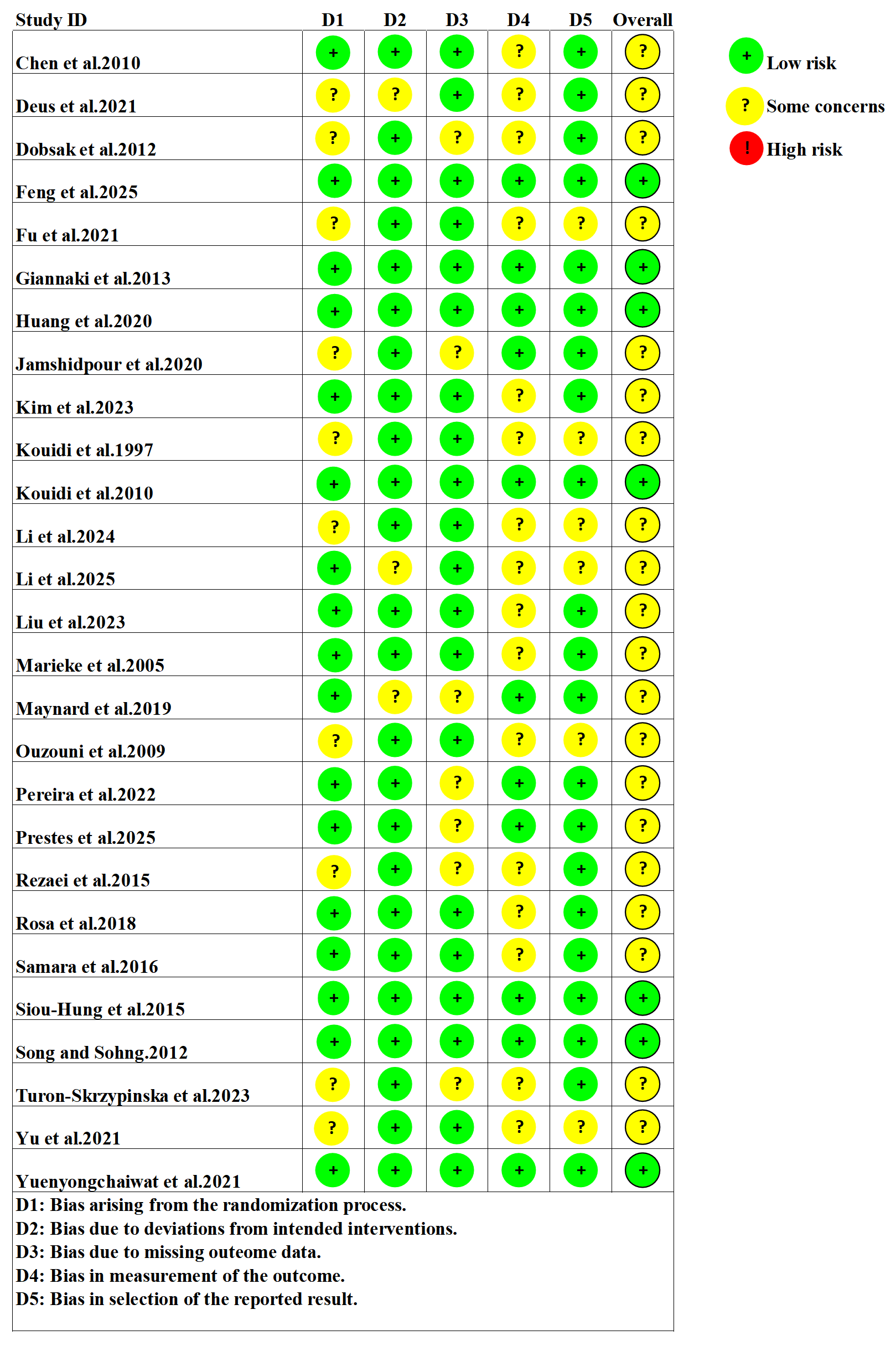


**Supplementary Figure 1.** Risk of bias assessment


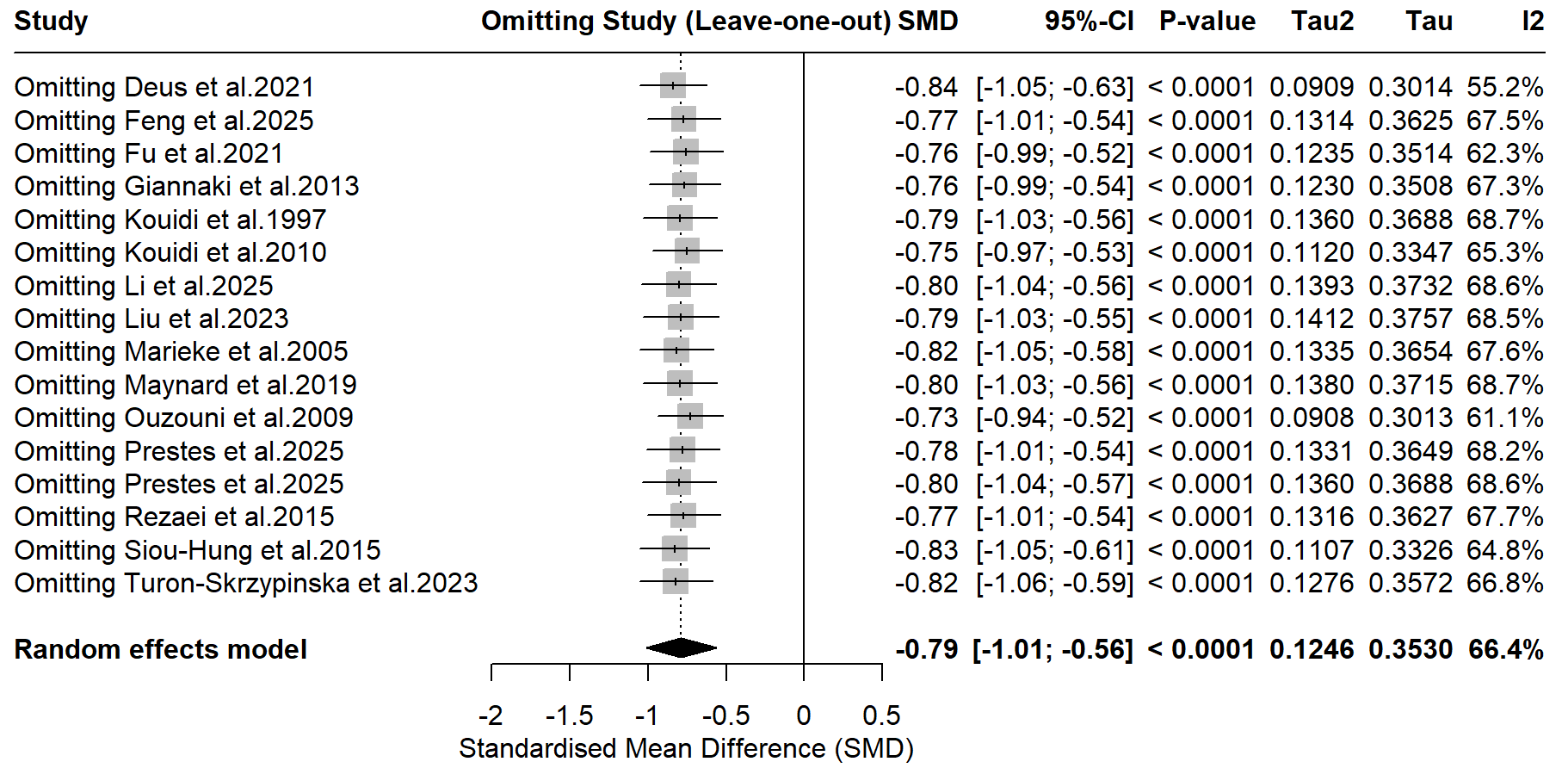


**Supplementary Figure 2.** Leave-one-out analysis of studies assessing depression severity in MHD patients


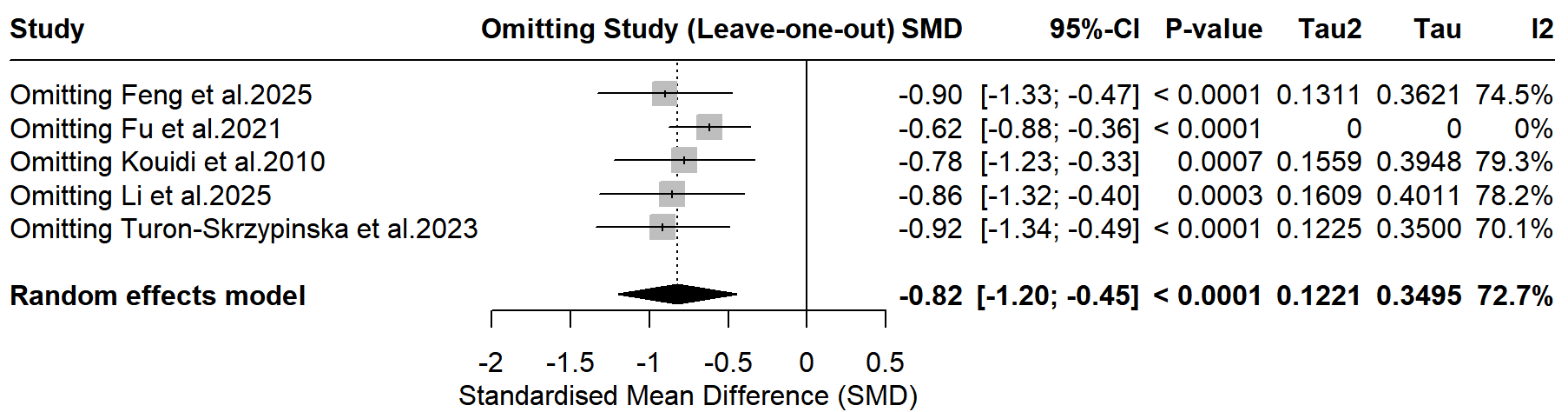


**Supplementary Figure 3.** Leave-one-out analysis of studies assessing anxiety severity in MHD patients


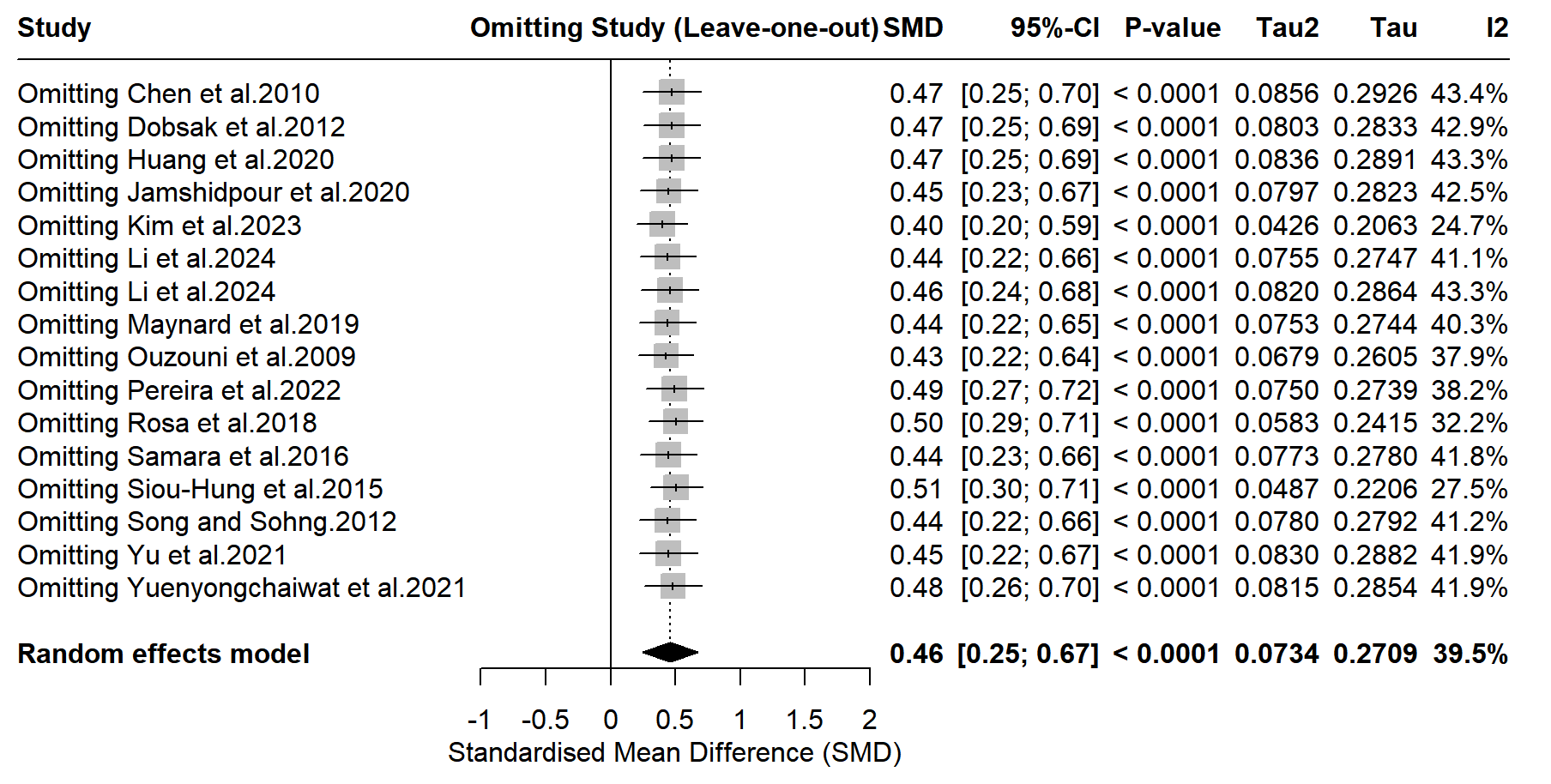


**Supplementary Figure 4.** Leave-one-out analysis of studies assessing physical health-related quality of life levels in MHD patients


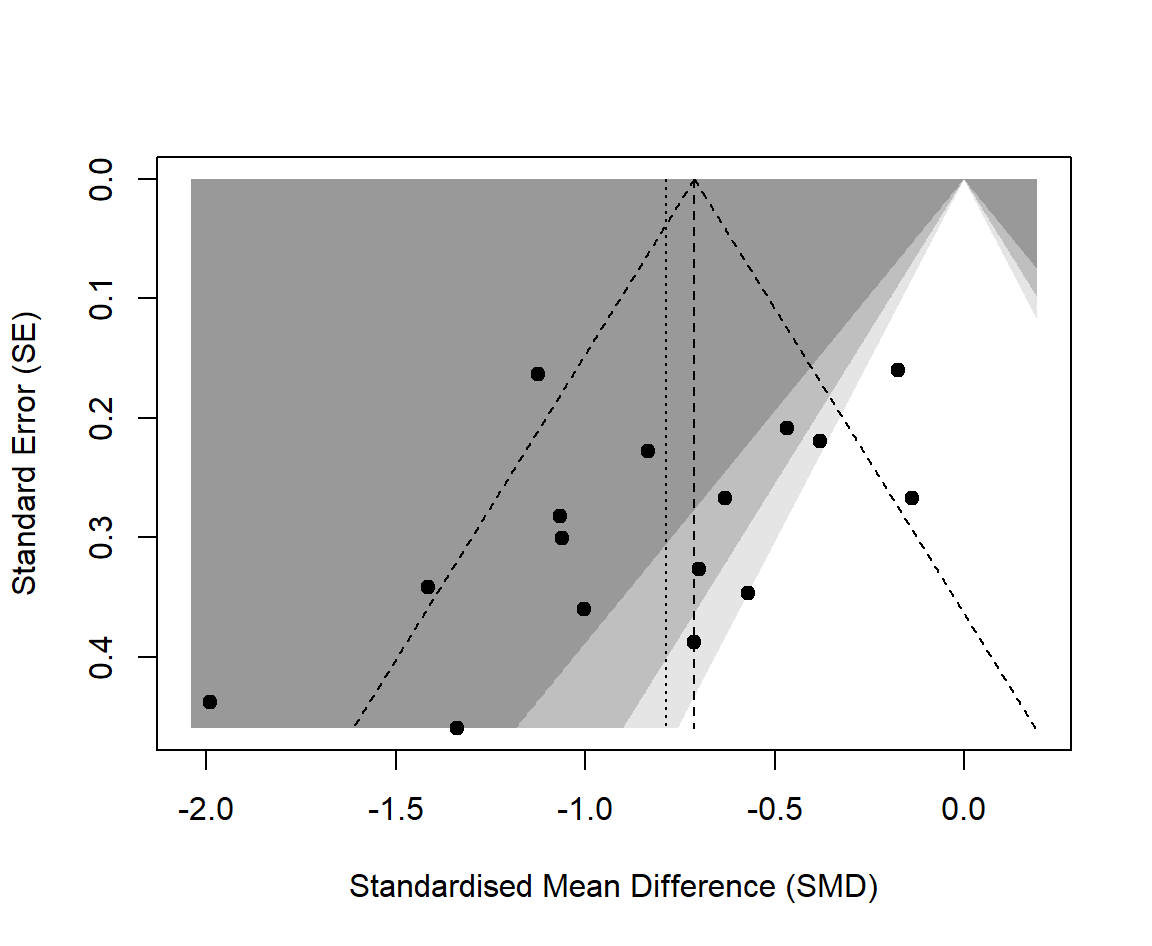


**Supplementary Figure 5.** Funnel plot for visual inspection of publication bias in studies assessing depression severity in MHD patients


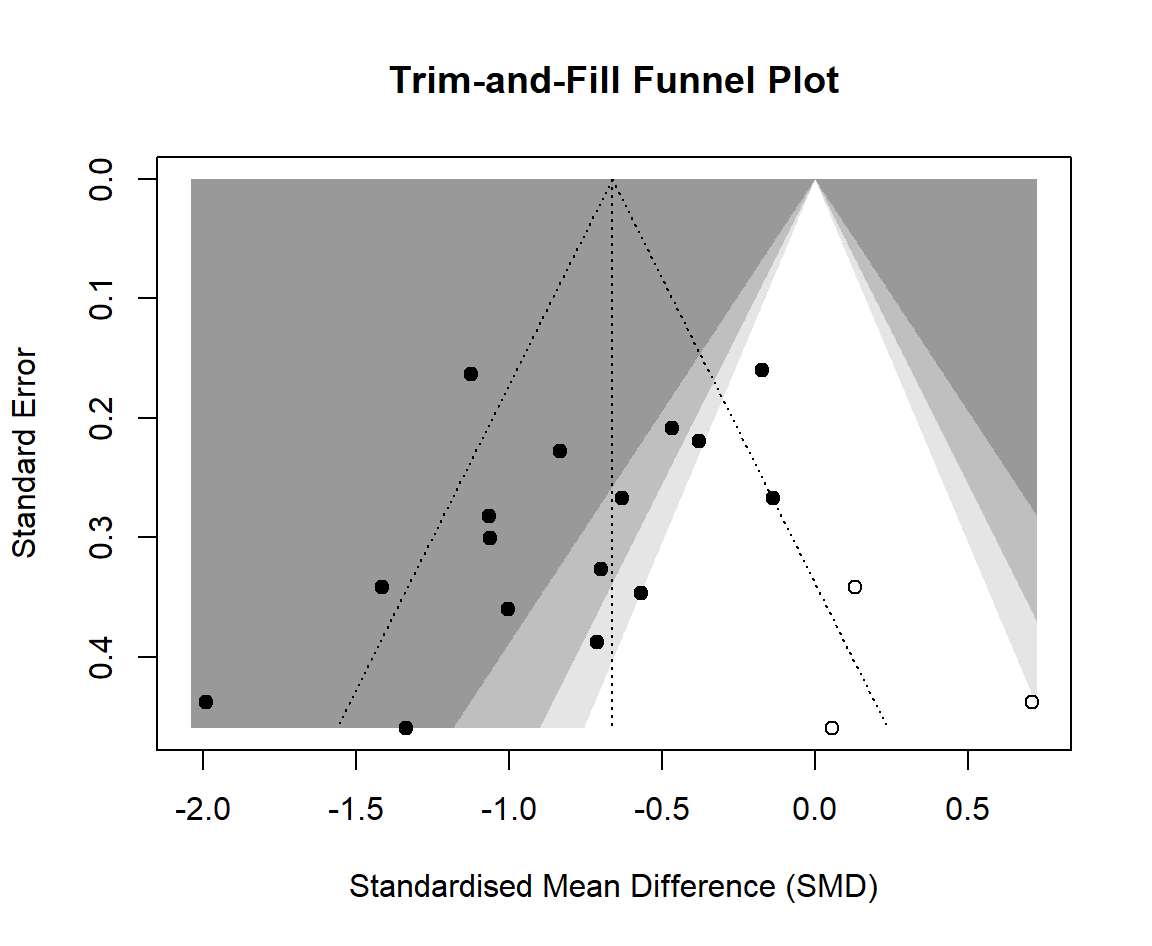


**Supplementary Figure 6.** Trim-and-fill analysis for publication bias in studies assessing depression severity in MHD patients


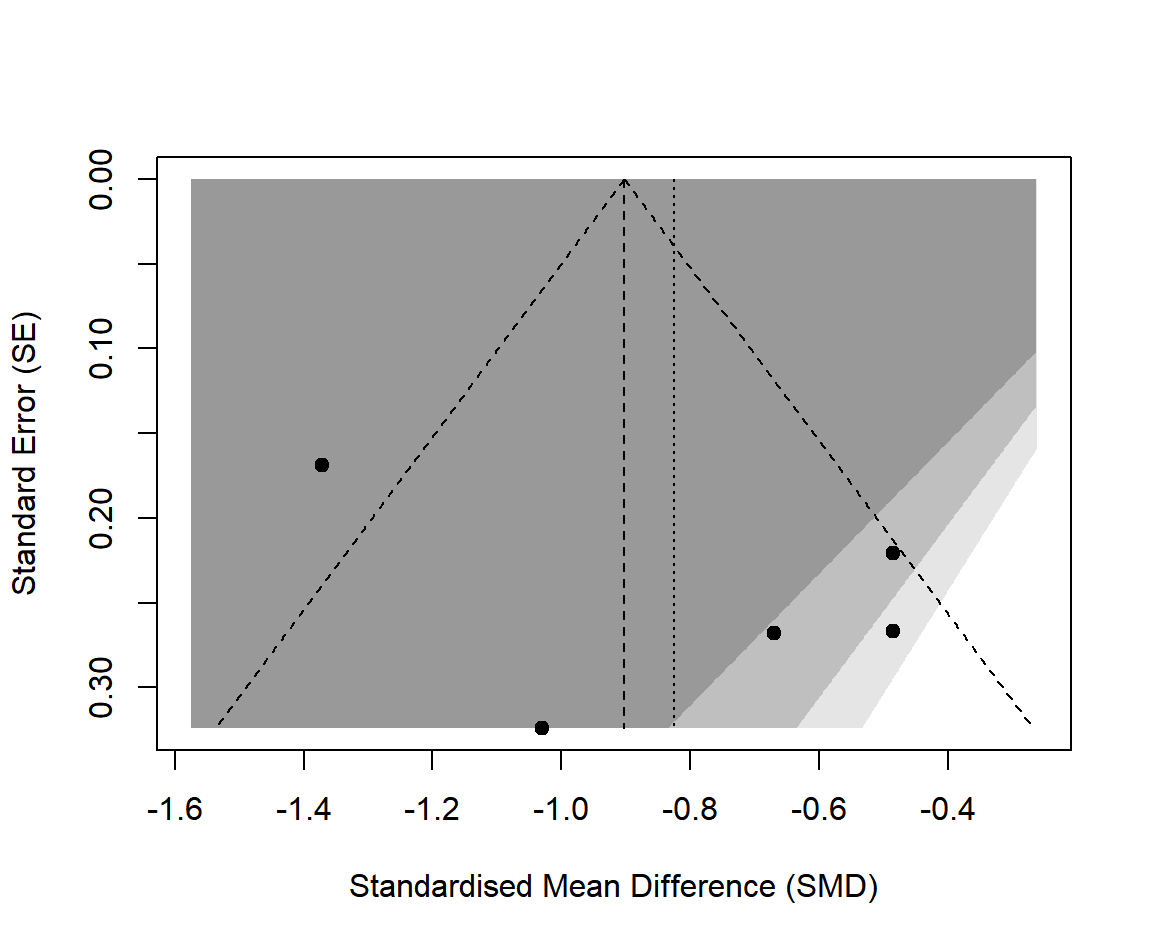


**Supplementary Figure 7.** Funnel plot for visual inspection of publication bias in studies assessing anxiety severity in MHD patients


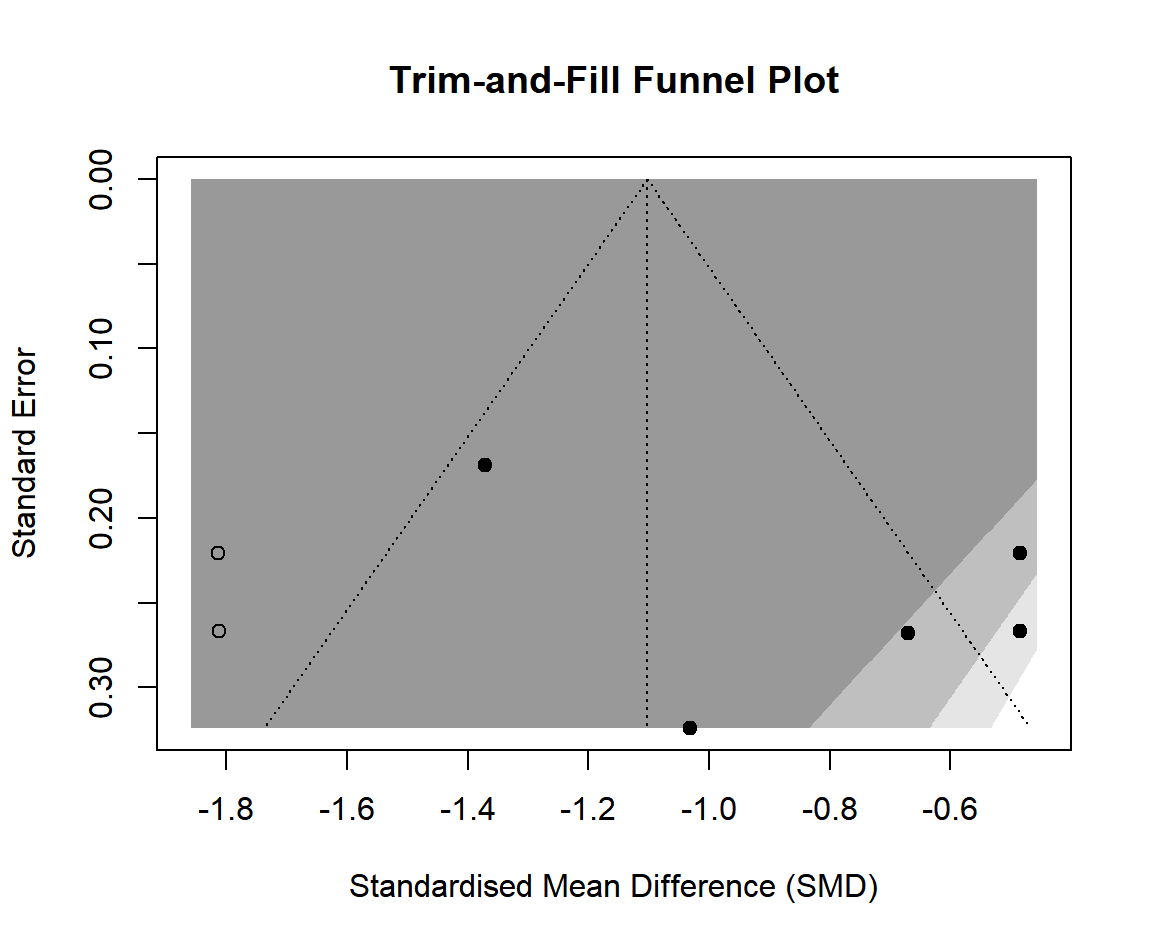


**Supplementary Figure 8.** Trim-and-fill analysis for publication bias in studies assessing anxiety severity in MHD patients


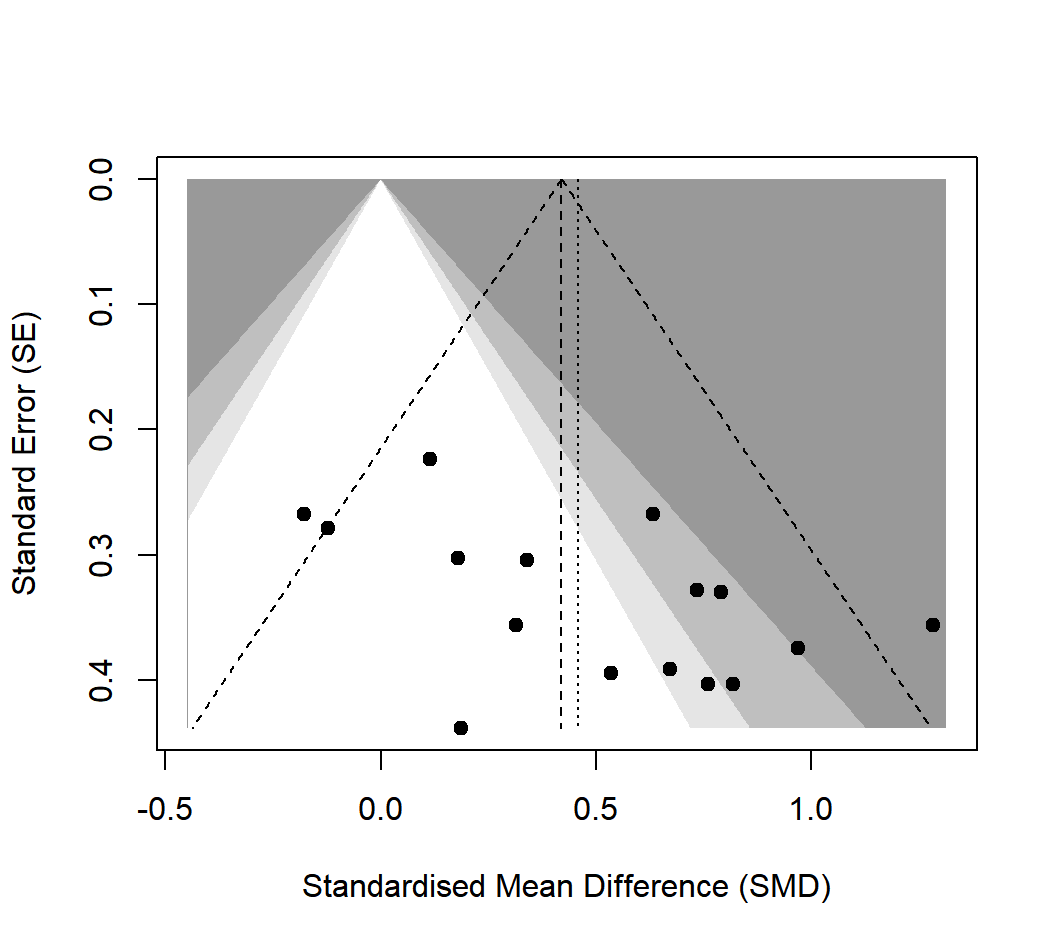


**Supplementary Figure 9.** Funnel plot for visual inspection of publication bias in studies assessing physical health-related quality of life levels in MHD patients


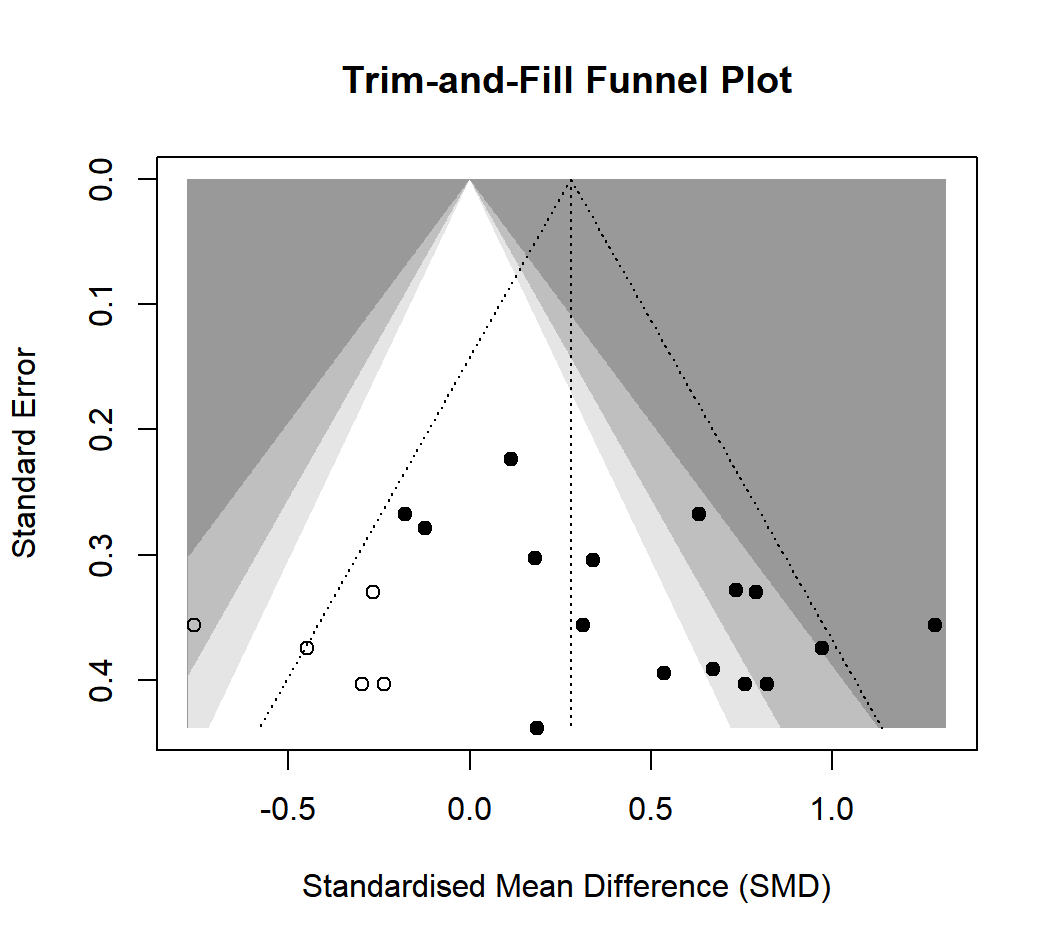


**Supplementary Figure 10.** Trim-and-fill analysis for publication bias in studies assessing physical health-related quality of life levels in MHD patients
